# Recombination directionality factor gp3 binds ϕC31 integrase via the zinc domain, potentially affecting the trajectory of the coiled-coil motif

**DOI:** 10.1093/nar/gkx1233

**Published:** 2017-12-08

**Authors:** Paul C M Fogg, Ellen Younger, Booshini D Fernando, Thanafez Khaleel, W Marshall Stark, Margaret C M Smith

**Affiliations:** 1 Department of Biology, University of York, Wentworth Way, York, YO10 5DD, UK; 2 Institute of Medical Sciences, Foresterhill, University of Aberdeen, Aberdeen AB25 2ZD, UK; 3 Institute of Molecular, Cell and Systems Biology, University of Glasgow, Glasgow G12 8QQ, UK

## Abstract

To establish a prophage state, the genomic DNA of temperate bacteriophages normally becomes integrated into the genome of their host bacterium by integrase-mediated, site-specific DNA recombination. Serine integrases catalyse a single crossover between an attachment site in the host (*attB)* and a phage attachment site (*attP)* on the circularized phage genome to generate the integrated prophage DNA flanked by recombinant attachment sites, *attL* and *attR*. Exiting the prophage state and entry into the lytic growth cycle requires an additional phage-encoded protein, the recombination directionality factor or RDF, to mediate recombination between *attL* and *attR* and excision of the phage genome. The RDF is known to bind integrase and switch its activity from integration (*attP x attB*) to excision (*attL x attR*) but its precise mechanism is unclear. Here, we identify amino acid residues in the RDF, gp3, encoded by the *Streptomyces* phage ϕC31 and within the ϕC31 integrase itself that affect the gp3:Int interaction. We show that residue substitutions in integrase that reduce gp3 binding adversely affect both excision and integration reactions. The mutant integrase phenotypes are consistent with a model in which the RDF binds to a hinge region at the base of the coiled-coil motif in ϕC31 integrase.

## INTRODUCTION

Temperate bacteriophages usually encode an integrase that mediates site-specific recombination between the host attachment site, *attB*, and the phage attachment site, *attP*, in the establishment of a lysogen. Phage integrases belong to one of two evolutionarily and mechanistically different families; the serine and the tyrosine integrases (1). Unlike tyrosine integrases, serine integrases mediate integration without the requirement for an accessory protein, and their attachment sites are short sequences of <50 bp (1–4). Recombination between *attP* on the circularized phage genome and the host *attB* site results in the integrated prophage, flanked by two new attachment sites, *attL* and *attR*, each containing reciprocal halves from *attP* and *attB*. While integrase alone can efficiently catalyse the *attP* ×*attB* integration reaction, it is inactive on the *attL* and *attR* sites (5,6), yet excision (i.e. *attL* ×*attR* recombination) is required so that the phage can re-enter the lytic cycle. A second phage-encoded protein, the recombination directionality factor (RDF) binds integrase and activates excision whilst inhibiting integration (7–9). Thus the site-specific recombination system in temperate phages is highly directional and controlled by both the nature of the recombination sites and the requirement for an RDF to switch the activity of integrase from its default activity (integration) to excision.

Integrases have been successfully used in a vast array of applications including chromosomal insertions and genome rearrangements (10). They are highly attractive tools for synthetic biologists and have been used to create binary genetic switches (11). When multiple orthogonal integrase systems are combined, biological computers that count and record stimuli can be constructed (12–14). A particular advantage of serine integrases is that they can be used for ordered or random assembly of multiple DNA fragments in a single, efficient *in vitro* reaction (15,16).

However, a limiting factor for the use of serine integrases for complex, multiplexed applications is the number of well-characterized integrases and, perhaps more pressingly, RDFs. Seven orthogonal integrase/RDF pairs have been identified to date (from phages ϕC31, Bxb1, TP901, ϕJoe, SPBc, ϕRv1 and A118) (7,8,17–21). While integrases are detected easily by their primary amino acid sequence similarities and their cognate attachment sites can often be predicted from comparative genomics at the prophage integration sites (14), RDFs are much harder to identify due to their diversity of size, genetic loci and amino acid sequence (18). A greater understanding of how the directionality switch works for integrases could enhance the applications of serine integrases.

The mechanism of integration has been studied extensively in a number of model systems (2). Using ϕC31 integrase, it was shown early on that although integrase could bind as a dimer to all four attachment sites, it could only generate a synaptic tetramer when bound to the *attP* and *attB* sites. Its inability to synapse *attL* and *attR* sites explained why integrase is recombinationally inactive for the excision reaction (6,22). Formation of a synaptic tetramer with *attP* and *attB* sites is rapidly followed by DNA cleavage with concomitant generation of covalent phosphoserine bonds between the four cleaved DNA ends and the four integrase subunits. Cleavage is followed by strand exchange through a subunit rotation mechanism and then the phosphodiester DNA backbones are ligated to form the recombinant products (23).

A significant advance in understanding the directional control of integrases came when a structural model of the large C-terminal domain (CTD) of a *Listeria innocua* (LI) prophage serine integrase bound to an *attP* half site was determined by X-ray crystallography (24,25). LI integrase is 98% identical to A118 integrase and they share highly similar attachment sites. The large CTD of LI integrase contains two DNA binding subdomains; the recombinase domain (RD) and the zinc domain (ZD). A coiled-coil motif is embedded within the ZD which has been shown to make protein-protein contacts between subunits (26,27). Structural models of the LI integrase show that these contacts form protein-protein interactions between two DNA bound integrase dimers in the formation of the tetrameric synaptic complex (inter-dimer contacts) and within an integrase dimer bound to the *attL* site (and by extrapolation to *attR;* intra-dimer contacts) (24,27). Intra-dimer coiled-coil interactions between subunits bound to *attL* and *attR* are thought to inhibit the formation of the tetrameric synaptic complex, explaining why integrases are inactive on *attL* and *attR* and only mediate *attP* × *attB* recombination in the absence of the RDF (24,27,28). These conclusions confirm previous proposals on the mechanism of ϕC31 integrase, notably that the isolated CTD (comprising both RD and ZD) can form tetrameric synaptic complexes when bound to *attP* and *attB* and that the CTD can bind to *attL* and *attR* cooperatively (26,29).

The RDF binds integrase and acts stoichiometrically to promote *attL* ×*attR* recombination (7,8,19). Recently, Mandali *et al.* proposed that the RDF for *Listeria* phage A118, gp44, acts by binding to the base of the coiled-coil motif, perhaps altering the trajectory of the motif and enabling the inter-dimer contacts required for formation of a synaptic complex (19,28). As RDFs are extremely diverse, a question remains as to whether this mechanism is general. The ϕC31 RDF, gp3, is unrelated to gp44 from A118, so we sought to identify the regions of gp3 and ϕC31 integrase that interact. We identify key amino acids in both ϕC31 integrase and gp3 that are required for the interaction between the two proteins. As expected, integrase mutants unable to bind gp3 are defective for excision, but they are also highly defective for integration. We propose that gp3 binds to a hinge region in ϕC31 integrase and that the flexible nature of this hinge is required for both integration and excision.

## MATERIALS AND METHODS

### Bacteria and plasmids

*Escherichia coli* DH5α (*fhu*A2 Δ(*arg*F-*lac*Z)U169 *pho*A *gln*V44 Φ80 Δ(*lac*Z)M15 *gyr*A96 *rec*A1 *rel*A1 *end*A1 *thi*-1 *hsd*R17) was used as a general cloning host. *E. coli* BTH101 (F-, *cya-99, araD139, galE15, galK16, rpsL1 (Str^R^), hsdR2, mcrA1, mcrB1*) was used for the bacterial two-hybrid (B2H) assays (30). *E. coli* strains were grown in LB or LB agar containing the appropriate antibiotics (ampicillin 100 μg/ml; chloramphenicol 50 μg/ml; hygromycin 100 μg/ml; kanamycin 50 μg/ml), IPTG (1 mM) and XGal (80 μg/ml) where appropriate. Plasmids used are shown in Table 1 and Supplementary Table S1. DNA oligonucleotides are shown in Supplementary Table S2.

**Table 1.** Plasmids

Plasmid	Description	Experiment	Antibiotic	Reference
pKT25	Vector	B2H	Kan	(30)
pUT18	Vector	B2H	Amp	(30)
pUT18C	Vector	B2H	Amp	(30)
pEHISTEV	T7 promoter-*his6* tag	Protein expression	Kan	(40)
pEY4	*tcp830* promoter-ϕC31 *int*	*In vivo* rec	Hyg	(8)
pEY120	pUT18, ϕC31 *g3*	*In vivo* rec	Amp	(8)
pARM152	pACYCDuet, ϕC31 *g3*	*In vivo* rec	Cm	This Study
pPAR1000	ϕC31 *attL^360^-lacZ-attR^475^*	*In vivo* rec	Kan	(29)
pLT27	ϕC31 *attB^373^-lacZ-attP^464^*	*In vivo* rec	Cm	(41)
pEY111	ϕC31 *int-t18* fusion	B2H	Amp	(8)
pEY110	*t18-*ϕC31 *int* fusion	B2H	Amp	(8)
pCMF18	*t25*- ϕC31 *g3* fusion	B2H	Kan	This Study
pARM010	*his6*-ϕC31 *int*	Protein expression	Kan	(32)
pEY301	*his6*-ϕC31 *g3*	Protein expression	Kan	(8)
pRT600	Contains *attB^50^*	*In vitro* rec	Amp	(42)
pRT702	Contains *attP^50^*	*In vitro* rec	Amp	(42)
pRT600702	Contains *attL^50^ & attR^50^*	*In vitro* rec	Amp	(42)
pTK33	*t25* - ϕBT1 *g3* fusion	B2H	Kan	This Study
pTK32	*t18* - ϕBT1 *int* fusion	B2H	Amp	This Study
pCMF30	*t25* - TG1 *g25* fusion	B2H	Kan	This Study

Plasmid pARM152 contains ϕC31 *g3* inserted into pACDuet1 (Novagen) and was constructed as follows: A PCR product generated using pEY301 as the template and primers (OARM110 and OARM111) was inserted into pACDuet1 cut with NcoI and HindIII by In-Fusion cloning to make pARM121, which was then cut with NcoI and the single-stranded 3′ ends digested with mung bean exonuclease to remove an unwanted ATG start codon. To transfer the mutated ϕC31 *g3* alleles from plasmids pARM152 or pEY120 to the B2H vectors (30), the *g3* alleles were amplified by PCR using C31 T25-gp3 F/R (pARM152 templates) or C31 T25-gp3 F/R2 (pEY120 templates) primers and sub-cloned into pKT25 cut with BamHI by In-Fusion cloning (Clontech). Similarly, for transfer of *g3* alleles to the expression vector, the *g3* genes were amplified with C31 H6-gp3 F/R primers (pARM152 templates) and inserted into pEHISTEV cut with NcoI and HindIII. Plasmid pCMF30 encodes TG1 gp25 and was constructed by generating a PCR product with TG1 genomic DNA and primers (TG1 T25-RDF F/R), which was inserted into pKT25 cut with BamHI by In-Fusion cloning. pTK32 encodes the T18 adenylate cyclase domain fused in frame to ϕBT1 integrase and was derived from pUT18C cut with PstI and BamHI ligated to a PCR product, also cut with PstI and BamHI, obtained using ϕBT1 DNA as the template and primers (TK89 and TK90). pTK33 contains ϕBT1 *g3* amplified by PCR from phage DNA (using primers EY44 and TK65) and inserted into the BamHI site of pKT25. Plasmids pEY110 and pEY114 to pEY118 were constructed by ligating PCR products (derived using ϕC31 *int* as the template and primers indicated in Supplementary Table S2) (cut with PstI and XbaI) into pUT18C (also cut with PstI and XbaI). All constructs were sequenced using Sanger sequencing to verify that no unwanted mutations were introduced.

### Mutagenesis

Hydroxylamine mutagenesis of pARM152 and pEY120 plasmid DNAs was carried out as described (31). Plasmid DNA (∼250 ng) was incubated in 1 M hydroxylamine (pH 6.7), final volume 12.5 μl, at 37°C for 20 h. The plasmids were then purified using a silica column (QIAGEN) prior to transformation into *E. coli* for screening via an *in vivo* recombination assay (see below). Clones that produced a phenotype of interest were sequenced to identify any nucleotide substitutions.

Error-prone PCR mutagenesis was carried out using Taq polymerase in standard reaction buffer (NEB) containing 1 ng template DNA (pEY111), 20 μM dGTP/dATP, 100 μM dCTP/dTTP, 5 mM MgCl_2_, 500 μM MnCl_2_, 3% DMSO, 5 U Taq polymerase and 0.2 μM C31 CTD F/R primers. Each reaction consisted of 30 cycles of 94°C/1 min, 50°C/1 min, 72°C/1 min. PCR products were used to replace the 743 bp NcoI-XbaI fragment from pEY111 (encoding the C-terminal region of ϕC31 integrase), and transformants were screened for loss of a B2H signal with partner plasmid pCMF18 encoding a T25-ϕC31 gp3 (wild type) fusion protein.

Site-directed mutagenesis was achieved by inverse PCR of 1 ng template DNA (pEY111, pEY4, pARM010 or pTK32) using overlapping primers (offset by 8–10 bp) containing the desired mutation (Supplementary Table S2). Phusion hi-fidelity polymerase (ThermoFisher) was used according to the manufacturer's recommendations with the following reaction conditions: 25 cycles of 98°C/10 s, 60°C/15 s, 72°C/3 min. PCR products were digested with DpnI restriction endonuclease overnight at 37°C and introduced without further treatment into chemically competent *E. coli* by transformation. Deletion of the ϕC31 integrase coiled-coil motif was achieved by inverse PCR of integrase-containing plasmids (pEY111, pARM010 and pEY4). The primer binding sites were located either side of the coiled-coil with 15 bp complementary 5′ tails. The resultant PCR products were then circularized using In-Fusion, DpnI treated and introduced into competent *E. coli* by transformation.

### Bacterial-two-hybrid (B2H) assays

The procedure and the resources were as described in (30). Plasmids encoding T18 (pUT18 or pUT18C and derivatives) and the compatible plasmids encoding T25 (pKT25 and derivatives) were introduced pairwise into competent BTH101 by co-transformation. Selection was using LB agar containing 50 μg/ml kanamycin, 100 μg/ml ampicillin, 1 mM IPTG and 80 μg/ml X-Gal, and plates were incubated at 30°C for 24–48 h. The phenotype of BTH101 (*cya*-) can be complemented if the two domains of adenylate cyclase (T18 and T25) are brought into close proximity, and this can be achieved by fusing interacting protein partners to each domain. The readout for complementation of the *cya*- phenotype (indicating a positive interaction between the two fusion proteins) is the induction of *lac* (blue colonies on IPTG, XGal), whereas no induction (white colonies) indicates no fusion protein interaction.

### Assay of β-galactosidase activity

Colonies obtained from the B2H plasmids introduced into BH101 were spotted onto selective agar. The confluent spots were used to inoculate 200 μl aliquots of LB supplemented with 50 μg/ml kanamycin, 100 μg/ml ampicillin and 1 mM IPTG in a 96-well plate. Plates were covered and incubated for 16 h at 30°C with agitation. Absorbance (OD_600_) readings were taken using a plate reader. In a second 96-well plate, 80 μl aliquots of permeabilization solution (100 mM Na_2_HPO_4_, 20 mM KCl, 2 mM MgSO_4_, 0.06% (w/v) CTAB, 0.04% (w/v) sodium deoxycholate, 0.0054% (v/v) TCEP) were prepared. 20 μl aliquots from each well of the cultured bacteria were added to the corresponding wells of the plate containing the permeabilization solution and the mixtures incubated at room temperature for 15 min. 25 μl of the permeabilized samples were then added to 150 μl of substrate solution (60 mM Na_2_HPO_4_, 40 mM NaH_2_PO_4_, 1 mg/ml ONPG and 0.0027% (v/v) TCEP) that had been placed in a third 96-well plate. Absorbance (OD_420_) readings were taken in the plate reader at 10 minute intervals over 60 min at 30°C. The maximum two-point slope was calculated (ΔOD_420_/min/ml).

### *In vivo* recombination assays

To assay intramolecular *attB* × *attP* recombination events *in vivo*, the reporter plasmid pLT27 (containing *attB* and *attP* flanking a *lacZα* gene) and pEY4 (encoding ϕC31 integrase) were introduced concomitantly into *E. coli* DH5α by co-transformation. DH5α (pLT27, pEY4) were plated on LB agar containing 50 μg/ml chloramphenicol and 100 μg/ml hygromycin for plasmid selection, and 80 μg/ml X-Gal and 1 mM IPTG to detect presence or absence of the *lacZα* gene. To assay intramolecular *attL* × *attR* recombination in *E. coli* the reporter plasmid pPAR1000 (containing *attL* and *attR* flanking *lacZα*), along with pEY120 (encoding gp3) and pEY4 were introduced concomitantly into *E. coli* by co-transformation and plated onto LB agar containing 100 μg/ml hygromycin, 50 μg/ml kanamycin, 100 μg/ml ampicillin, 80 μg/ml X-Gal and 1 mM IPTG. Recombination efficiencies were calculated as number of white colonies/total number of colonies.

### Protein purification

Proteins were purified as previously described (8,26). Briefly, 500 ml cultures of *E. coli* BL21 containing the relevant expression plasmid were induced at mid-exponential growth phase with 0.15 mM IPTG overnight at 17°C. Concentrated cells were lysed in 20 ml binding buffer (1 M NaCl, 75 mM Tris pH 7.75) plus 0.2 mg/ml lysozyme and 500 U Basemuncher Endonuclease (Expedeon Ltd.) for 30 min on ice and then sonicated. Cleared supernatant was applied to a 5 ml HisTrap FF crude column (GE Healthcare) and the bound, his-tagged protein was eluted with 125 mM imidazole. Eluted protein was desalted on a HiPrep 26/10 desalting column (GE Healthcare) and then further separated by size exclusion chromatography on a HiLoad 16/60 Superdex 200 preparative grade gel filtration column. Purified proteins were concentrated in a Vivaspin sample concentrator (GE Healthcare) and quantified (Nanodrop, Thermo Scientific). Samples were stored at –80°C in binding buffer plus 50% glycerol.

### *In vitro* recombination assays

Assays were performed as described previously (32). Briefly, 20 μl reaction mixtures contained two DNA substrates (each 100 ng) for *attB* × *attP* recombination (*attB* and *attP* in pRT600 and pRT702, respectively), or a single substrate containing both *attL* and *attR* (pRT600702), in RxE buffer (working concentration: 10 mM Tris pH 7.5, 100 mM NaCl, 5 mM DTT, 5 mM spermidine, 4.5% glycerol, 0.5 mg/ml BSA), and the stated concentrations of integrase with or without gp3. Reactions were incubated for 1 h at 30°C, the integrase was then deactivated for 10 min at 75°C and the plasmids linearized by restriction digest with HindIII (NEB). The reaction mixes were then run on a 0.8% (w/v) agarose gel and band intensities measured using FIJI (NCBI). Recombination efficiencies were calculated as intensity of product band(s)/sum intensity of all bands.

### Modelling of ϕC31 C-terminal domain 3-dimensional structure

Phyre2 matches the Hidden Markov Models of the query sequence against the HMMs of the 3D models in the structural databases (33). Phyre2 was used to produce a 3D model of the C-terminal domain of ϕC31 integrase (aa 153–605) and was also used in one-to-one threading of just the ZD domain of ϕC31 integrase (aa 328–605) against the LI integrase structure (4KIS-chainA). The models were visualized using PyMol (PyMOL v1.8.2.3; Schrödinger LLC).

## RESULTS

### Mutants of gp3 that cannot activate excision are defective in Int binding

**Figure 1.**
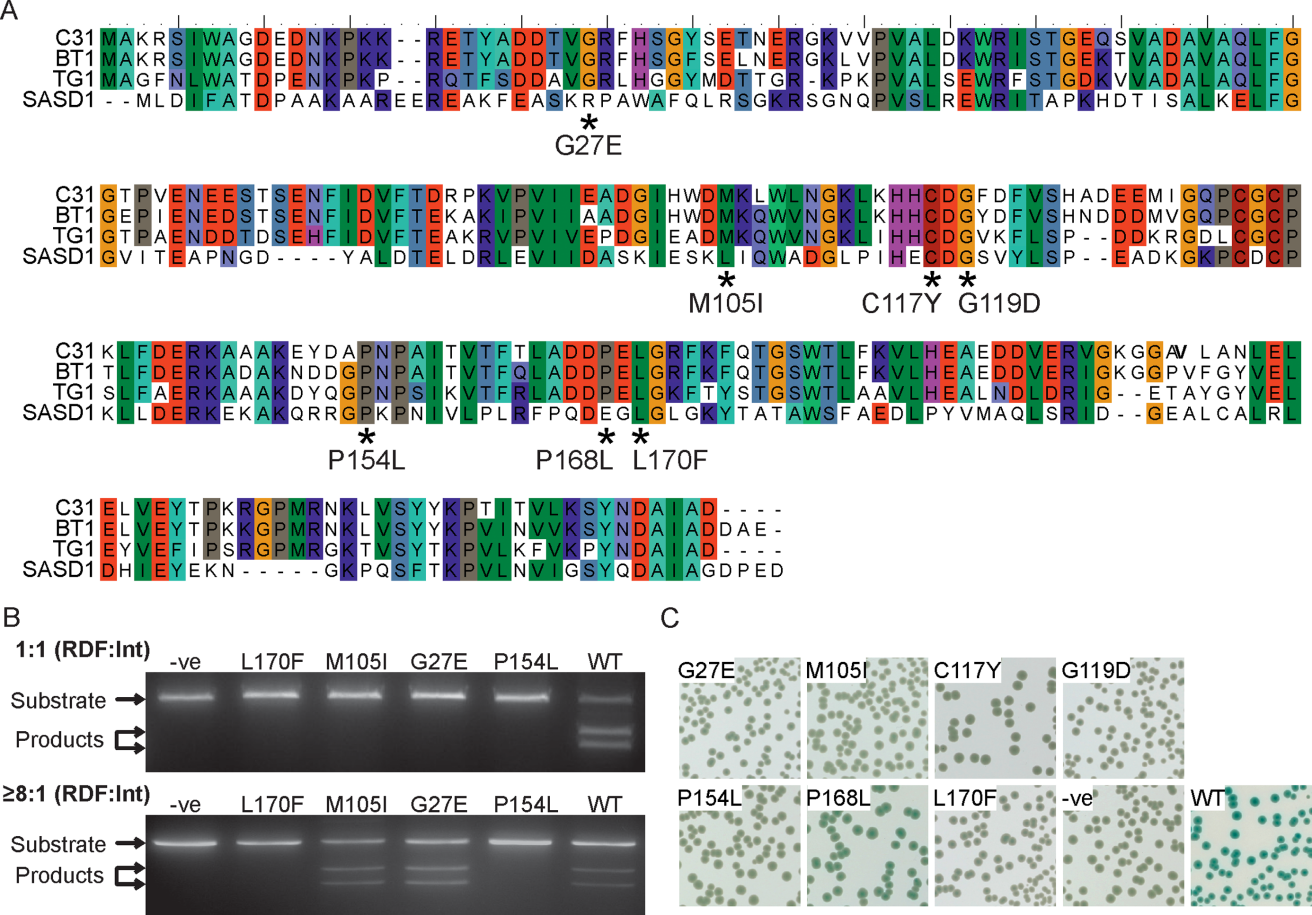
Mutational analysis of the ϕC31 RDF, gp3. (**A**) ClustalW alignment of proteins encoded by intact phage that are related to the ϕC31 RDF protein. Mutations identified that affect RDF function and interaction with the integrase are indicated by asterisks beneath the alignment. (**B**) Representative images of *in vitro* excision assays using wild-type ϕC31 integrase protein (200 nM) and either wild-type gp3 protein (WT) or gp3 with single amino acid substitutions, as indicated above the gels (gp3L170F, gp3M105I, gp3G27E and gp3P154L). The upper gel shows excisive recombination with a 1:1 ratio of gp3 to Int and the lower gel shows excision when gp3 is provided in excess (≥8:1 ratio). (**C**) Bacterial-two-hybrid assay of *E. coli* BTH101 strains containing wild-type ϕC31 integrase fused to T18 (encoded by pEY111) and T25 fused to wild-type gp3 (pCMF18) or gp3 mutants (pCMF7, pCMF8, pCMF9, pCMF11, pCMF12, pCMF19 and pCMF20 encoding gp3L170F, gp3M105I, gp3G119D, gp3G27E, gp3P154L, gp3C117Y and gp3P168L, respectively). Empty plasmids (pUT18 and pKT25) were used as a negative control (–ve).

Our initial objective was to question whether mutations in ϕC31 gp3 that failed to activate *attL* ×*attR* recombination correlated with reduced binding to ϕC31 integrase. If this was the case it would confirm that binding of gp3 to integrase is necessary for activation of excision. Plasmids encoding ϕC31 gp3 (either pARM152 or pEY120; Table 1) were mutagenized with hydroxylamine (Rose and Fink, 1987). The mutant plasmid libraries were then used in an *in vivo* intramolecular excision assay together with a plasmid (pEY4) encoding ϕC31 integrase and a reporter plasmid (pPAR1000) containing *lacZα* flanked by the ϕC31 *attL/attR* sites (Table 1). White colonies indicate that recombination has occurred, leading to loss of *lacZα*, whereas blue colonies indicate low or no recombination. Nineteen light blue or blue colonies were isolated, the gp3-expressing plasmid from independent isolates extracted and the assay repeated to check their inability to activate intramolecular excision. The *g3* genes (*g3* encodes gp3) were sequenced to identify mutations. Four plasmids contained nonsense mutations in *g3*, five had wild-type *g3*, one yielded poor sequence and was uninterpretable and the remaining nine had missense mutations in *g3* (including two pairs of duplicates). Thus we obtained seven unique mutations that were unable to activate intramolecular excision (Figure 1A). Four of the mutant *g3* alleles were transferred to an expression plasmid and the RDFs purified. In an intramolecular excision assay using a 1:1 ratio of gp3 to Int, no excision was detected (Figure 1B). However, two of the mutants (gp3M105I and gp3G27E) were able to promote excision when added in large excess over the integrase (Figure 1B), suggesting that these mutants had reduced integrase binding affinity.

Each of the seven *g3* alleles was transferred to the bacterial-two-hybrid (B2H) vector pKT25 and used for assay against wild-type integrase inserted into pUT18 (pEY111; Table 1). In the B2H assay a positive readout is the expression of the *lac* operon, activated by the cAMP whose synthesis is dependent on the two domains of adenylate cyclase that are brought together by the gp3-Int interaction. Previously, B2H analysis of pEY111 (encoding an Int-T18 fusion) and pEY85 (encoding a T25-gp3 fusion) yielded blue *E. coli* BH101 colonies on plates containing IPTG and XGal indicative of a positive interaction (8). All of the mutations in *g3* that abolished excision were also impaired for integrase binding; six out of seven alleles produced no visible blue at all and one (encoding gp3P168L) produced intermediate pale blue colonies, indicating reduced binding (Figure 1C).

There are only three verified, intact phage in GenBank encoding homologues to ϕC31 gp3; ϕBT1 gp3, TG1 gp25 and ϕSASD1 gp6 (34). All of the amino acids residues identified by mutagenesis are conserved in ϕBT1 gp3 and TG1 gp25, and five out of seven residues are similar in ϕSASD1 (Figure 1A). An extended alignment of ϕC31 gp3 with the three phage sequences above plus the 10 closest Blastp hits to prophage sequences, confirmed that the amino acids identified here are highly conserved (Supplementary Figure S1). Five of the seven amino acids examined (gp3G27, gp3C117, cp3G119, gp3P154 and gp3L170) were identical in >90% of proteins and one, although only 57% identical, was similar in 100% of proteins (gp3M105 or L). The amino acid with least conservation (gp3P168, 50% identity and similarity) also had the weakest impact on RDF-Int binding (Figure 1C). The high level of conservation of the gp3 residues important for integrase–RDF interaction implies that the residues could be part of a conserved structural fold that binds integrase. The amino acids identified may directly bind to integrase or they may alter the structural or physical properties of the binding site.

### Gp3 binds to the C-terminal 200 amino acids of ϕC31 integrase

**Figure 2.**
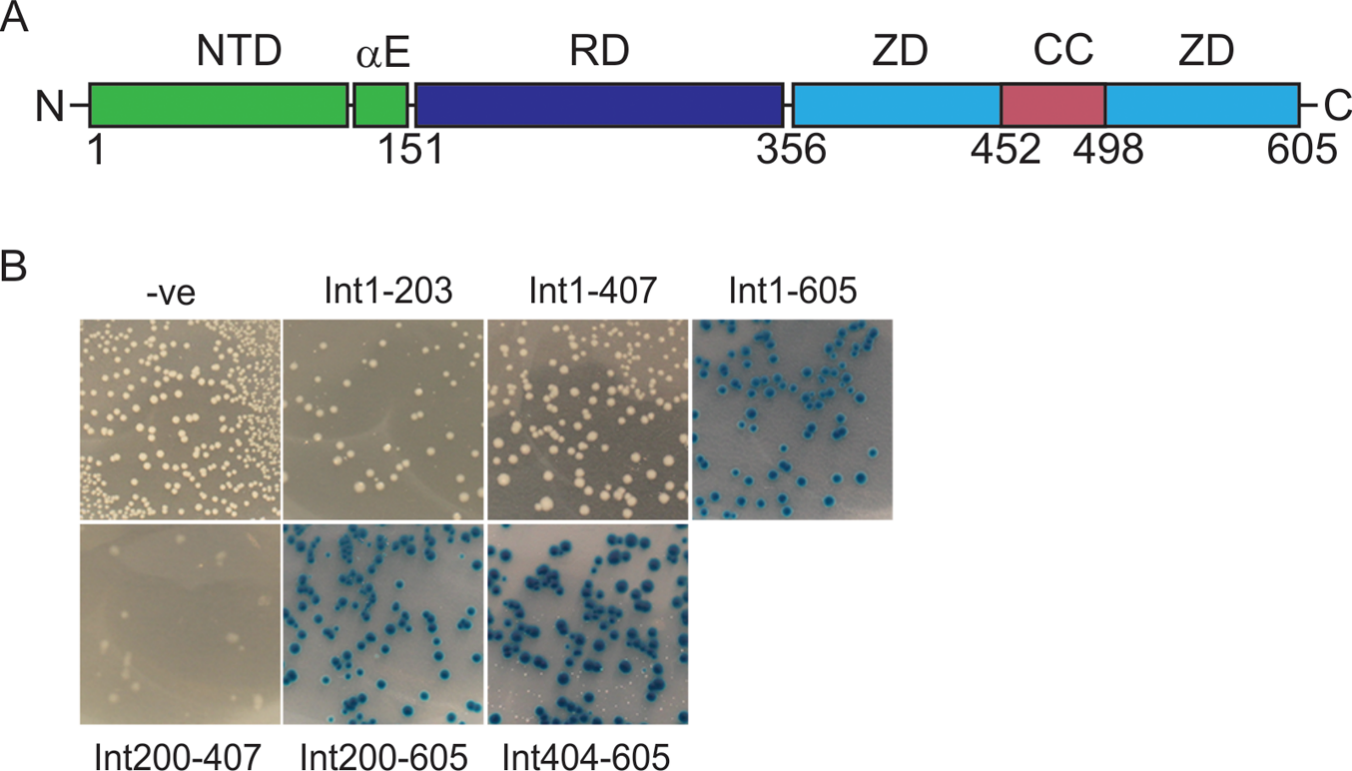
Localization of the gp3 interaction domain of integrase by bacterial-two-hybrid assays. (**A**) Schematic of the domain structure of ϕC31 integrase with approximate start and end coordinates indicated. NTD includes the N-terminal domain and the long αE helix (green). RD is the recombinase domain (purple). ZD is the zinc domain (blue) and this domain contains embedded within it the coiled coil (CC) motif (red). (**B**) Fragments of integrase were fused to T18 in pUT18C and these plasmids were used in B2H assays with pEY85 expressing T25-gp3 fusion (pEY85). Plasmids used contained fusions with full length integrase (amino acids (aa) 1–605; pEY110), aa 1–203 (pEY114), aa 1–407 (pEY115) aa 200–605 (pET116), aa 404–605 (pEY117), aa 200–407 (pEY118). The negative control (–ve) was pKT25.

We employed the B2H assay to localize the region of integrase that interacts with gp3 by cloning *int* gene fragments into one of the B2H vectors (pUT18C) to test for a positive interaction (Figure 2). Only T18C fusions containing the C-terminal 200 amino acids could still interact with gp3; this region contains most of the ZD domain that includes the coiled-coil motif.

### Isolation of integrases containing mutations that fail to bind gp3

**Figure 3.**
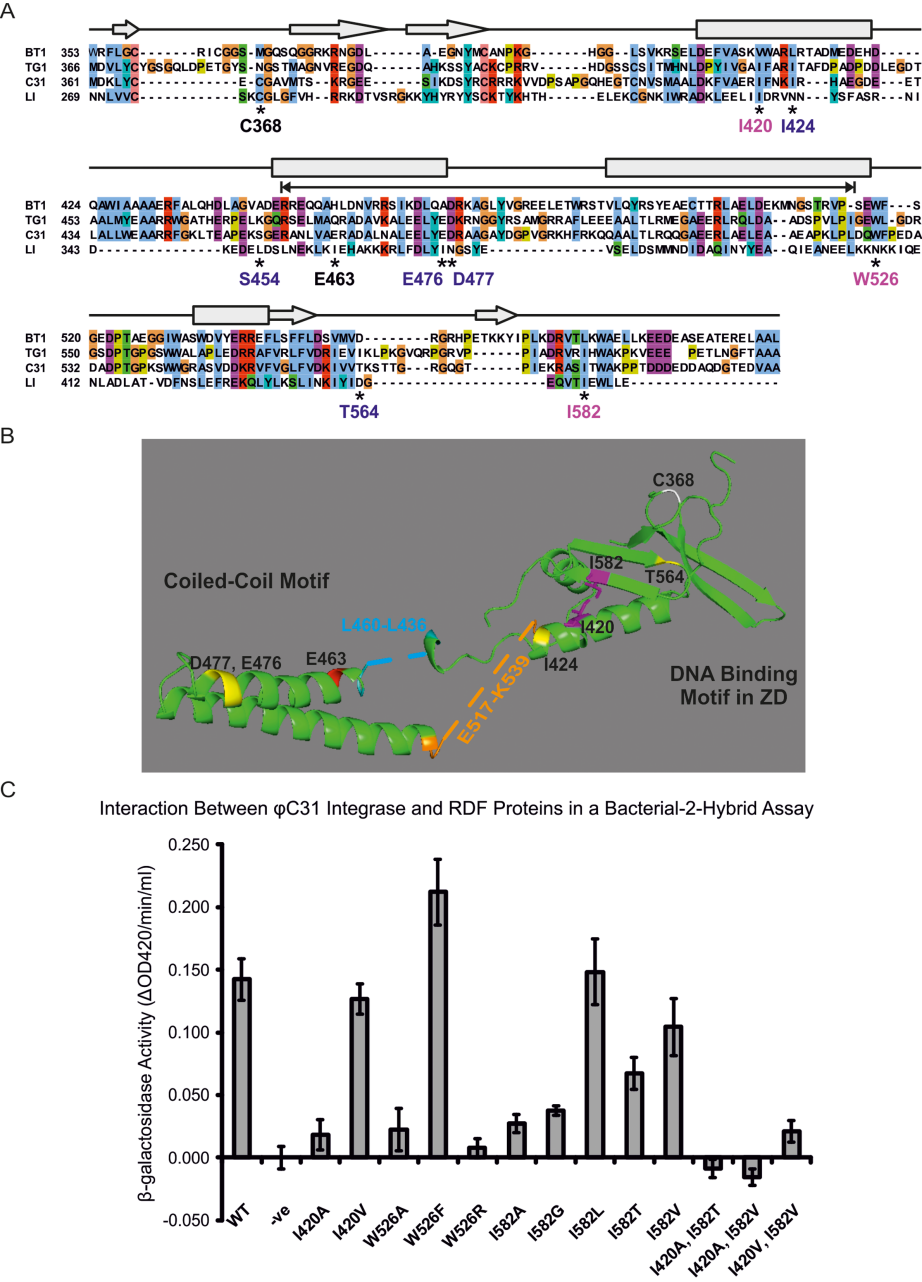
Mutational analysis of the ϕC31 integrase. (**A**) ClustalW alignment of ϕC31 integrase protein C-terminus (residues from 361 onwards) with related sequences. The predicted secondary structure based on A118 integrase (LI) (24) is represented above the alignment with boxes indicating α-helices and arrows indicating β-sheets. Mutations discussed in this article are indicated by asterisks and labels beneath the alignment; labels in magenta are locations where substitutions affect integrase function and interaction with the RDF, blue labels are where substitutions have no effect, and black indicates a previously described hyperactive mutant (E463K) and one of the cysteine residues (C368) that is thought to coordinate the Zn^2+^ ion. The residues removed for the coiled-coil deletion are indicated by a double-headed arrow. (**B**) Model of the zinc domain of ϕC31 integrase (amino acids 361 to 605) based on the LI integrase structure derived using Phyre2 (33). The structure contains two regions that were too disordered for prediction, indicated by blue and orange dashed lines and text. These regions contain the amino acid residues S454 and W526 respectively. The remaining mutations referred to in the text are labelled; magenta I420 and I582; yellow I424, E476, D477 and T564; red is the position of an E463K substitution that leads to hyperactivity (29); one of the cysteine residues (C368) that is thought to coordinate the Zn^2+^ ion is shown in white. (**C**) Quantification of the B2H signal by β-galactosidase assay. β-galactosidase activity is calculated as the change in absorbance at 420 nm per minute per ml. Integrase mutants are identified on the X-axis, and wild-type RDF was used in all cases.

Next we sought to fine-tune the region of integrase that interacts with gp3 by screening for amino acid substitutions that would abolish this interaction. We introduced random mutations by error-prone PCR in the NcoI to XbaI fragment of *int* in pEY111, which encodes the C-terminal 245 amino acids (aa 361–605). The mutagenized library was used to screen for colonies showing reduced binding between the Int-T18 and T25-gp3 fusion (pCMF18) in a B2H assay. White or pale blue colonies were picked, the pEY111-derived plasmid was purified and the *int* allele was sequenced. The mutagenesis procedure yielded more than one substitution per clone. To deconvolute which mutations conferred reduced binding, the individual mutations were introduced into *int* (in pEY111) by site-directed mutagenesis (Supplementary Table S2). Two substitutions in integrase were identified that conferred a reduced B2H signal; W526R and I582T (Figure 3A).

To gain an insight into how these substitutions might affect function, we used the structural model of *Listeria* phage LI (PDB ID: 4KIS) and alignments of the primary sequence of LI integrase with ϕC31 integrase (Figure 3A) (24,27). A 3D model of the C-terminal domain of ϕC31 integrase was also built using the modelling tool, Phyre2 (Figure 3B) (33). Both of the RDF non-binding mutations lay downstream of the predicted coiled-coil motif in integrase.

The integrase residue I582 is predicted to lie in a region of β-sheet, equivalent to LI integrase β14 (24). A variety of substitutions at I582 were generated by site-directed mutagenesis. IntI582A and IntI582G were both more severely affected in gp3 binding than the original IntI582T substitution, whereas both of the conservative changes I582L and Int I582V had little effect on binding to gp3 (Figure 3C), suggesting that a hydrophobic residue is important at this position. Although it contains a polar hydroxyl, the threonine substitution in IntI582T has the bulk and the methyl group that might substitute better for isoleucine than alanine or glycine. The 3D model of integrase from Phyre2 suggested that residue I582 could be interacting with another hydrophobic residue, I420, which is predicted to be within an α-helix (equivalent to LI αJ; (24)) (Figure 3). To test the importance of this residue in the ZD we hypothesized that substitutions in I420 might also affect gp3 binding. IntI420A but not IntI420V was defective in binding gp3, suggesting that there is a need for a hydrophobic amino acid in this region (Figure 3C). Although IntI420V could bind gp3 almost as well as wild-type integrase, introduction of second site substitutions at I582 generated double mutants that were severely affected in binding of gp3 (Figure 3C). The double mutant IntI420V, I582V was the least affected. A nearby isoleucine, I424, was also substituted with phenylalanine or alanine. Neither mutation had a significant impact on either integration or excision *in vivo*, suggesting that the specific location of the I420 residue is important. Lying anti-parallel to the ϕC31 equivalent of β14 is another predicted β-sheet, equivalent to LI β13 (Figure 3B). A T564A substitution here had no effect on the gp3 interaction, and IntT564A appeared as wild-type in the B2H assay. Substitutions in integrase residues I582 and I420, therefore, appear to disrupt the integrase-gp3 binding site by either altering its overall structure or by removing a specific binding interface for gp3.

Another substitution identified by random mutagenesis was IntW526R, which lies within a region of poor primary sequence similarity with LI integrase. This region in the Phyre2 model is highly disordered. However, W526 is predicted to be located at the C-terminal end of the coiled-coil motif. The chemistry of this residue was probed by generating further site-directed mutations at this position. IntW526A and the original mutant, IntW526R, were both defective in their interaction with gp3 whereas IntW526F, with a conservative large bulky hydrophobic R group had a stronger interaction with gp3 (Figure 3C). It is likely that this residue needs to be hydrophobic to enable integrase to interact with gp3. An integrase mutant that lacks the coiled-coil motif (aa 457 to 523 deleted) was unable to interact with gp3 in the B2H assay suggesting that the context of W526 is important for gp3 binding.

Other substitutions were introduced into the coiled-coil motif to test the interaction with gp3. IntS454G has a substitution at the N-terminal base of the coiled-coil, IntE476G and IntD477V both have amino acid changes close to the predicted turn in the coiled-coil; all three mutants were indistinguishable from wild-type integrase in their ability to interact with ϕC31 gp3 in the B2H assay (Figure 3C). Overall these studies suggest that there is a patch at the base of the coiled-coil motif in ϕC31 integrase that binds gp3 and that three of the residues involved need to be hydrophobic in nature. Mandali *et al.* also found that the coiled-coil motif was required for the interaction between A118 integrase and its RDF gp44 (19) but they did not identify specific residues.

### ϕC31 gp3 interacts with homologous regions in ϕBT1 and TG1 integrases

**Figure 4.**
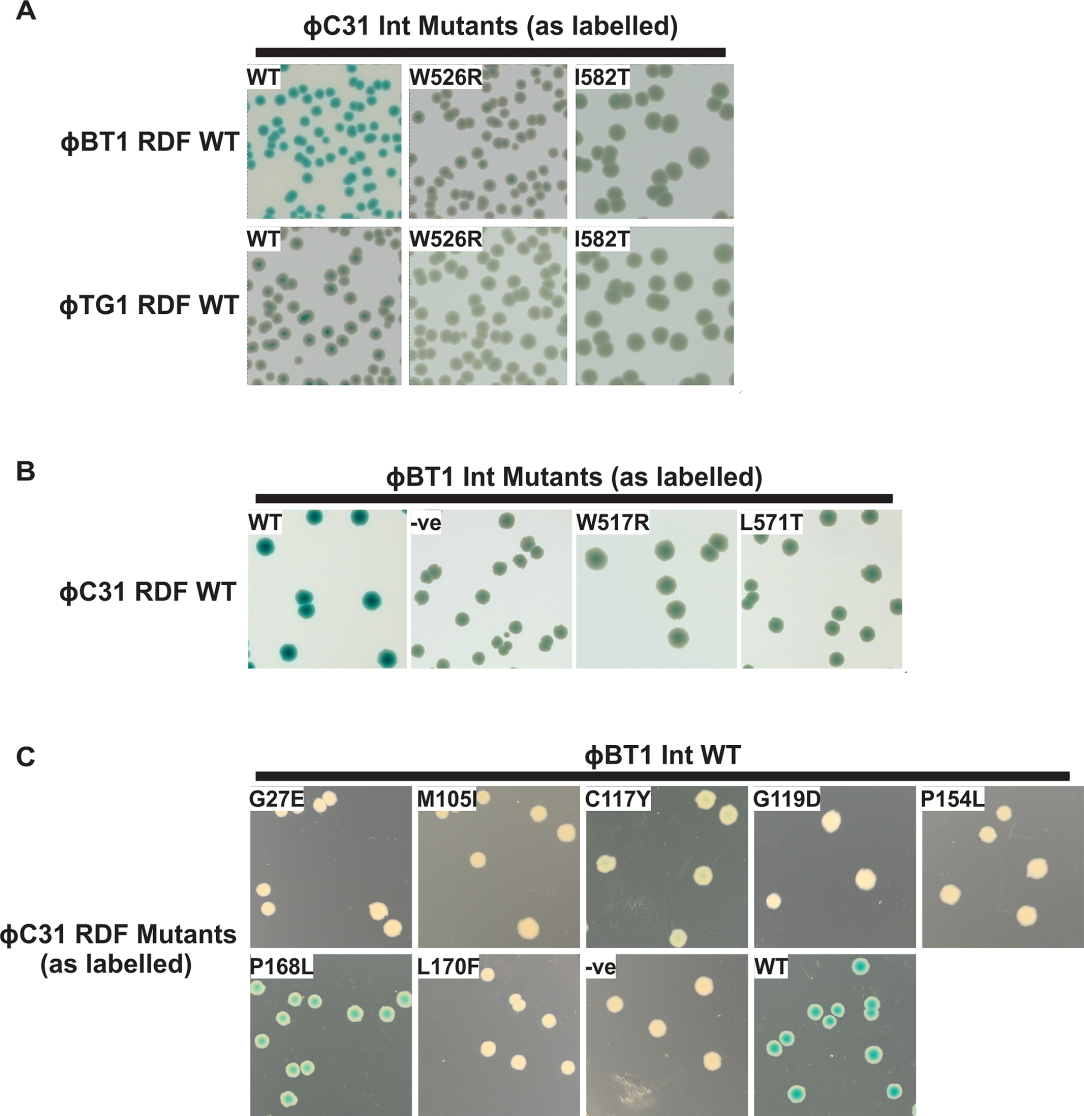
Bacterial-two-hybrid assays for Integrase-RDF binding *in vivo*. (**A**) ϕC31 integrase variants as indicated on each image (pEY111: wild-type, pCMF37: W526R or pCMF39: I582T) were assayed against wild-type ϕBT1 (plasmid pTK33, upper panels) and ϕTG1 (plasmid pCMF30, lower panels) RDFs. (**B**) ϕBT1 integrase mutants as indicated (pCMF120: W517R, pCMF121: L571T) were assayed against wild-type ϕC31 RDF (pCMF18). (**C**) Wild-type ϕBT1 integrase (pTK32) was assayed against ϕC31 RDF mutants as indicated (pCMF7: L170F, pCMF8: M105I, pCMF9: G119D, pCMF11: G27E, pCMF12: P154L, pCMF19: C117Y, pCMF20: P168L).

Phages ϕC31, ϕBT1 and TG1 are highly related (34); all three phages encode serine integrases and homologues of ϕC31 gp3. Indeed ϕC31 gp3 can act as an RDF for ϕBT1 integrase (35). As the amino acid residues required for the interaction of ϕC31 integrase with ϕC31 gp3 are conserved (Figure 3A), we questioned whether the same residues might be important in the TG1 and ϕBT1 homologues. In a B2H assay ϕC31 integrase is able to interact with ϕBT1 and TG1 RDFs, though the signal for TG1 RDF is considerably weaker than for the ϕC31 or ϕBT1 RDFs (Figure 4A). The ability of ϕC31 IntW526R and IntI582T mutants to interact with ϕBT1 or TG1 RDFs was impaired (Figure 4A). Conversely, comparable mutations to ϕC31 IntW526R and IntI582T were introduced into ϕBT1 Int (Int^ϕBT1^W517R and Int^ϕBT1^L571T), and both caused defective recognition of the ϕC31 gp3 in the B2H assay (Figure 4B). In addition, all of the ϕC31 gp3 mutants had substantially impaired binding with wild-type ϕBT1 integrase and, similar to ϕC31 integrase, only gp3P168L gave a moderately positive signal (Figure 4C). These data indicate that ϕC31 gp3 is likely to bind to the equivalent regions in both ϕBT1 and ϕC31 integrases, even though these two proteins only share about 24% identical residues.

### Integrase mutants that fail to bind gp3 are generally defective in both integration and excision

**Figure 5.**
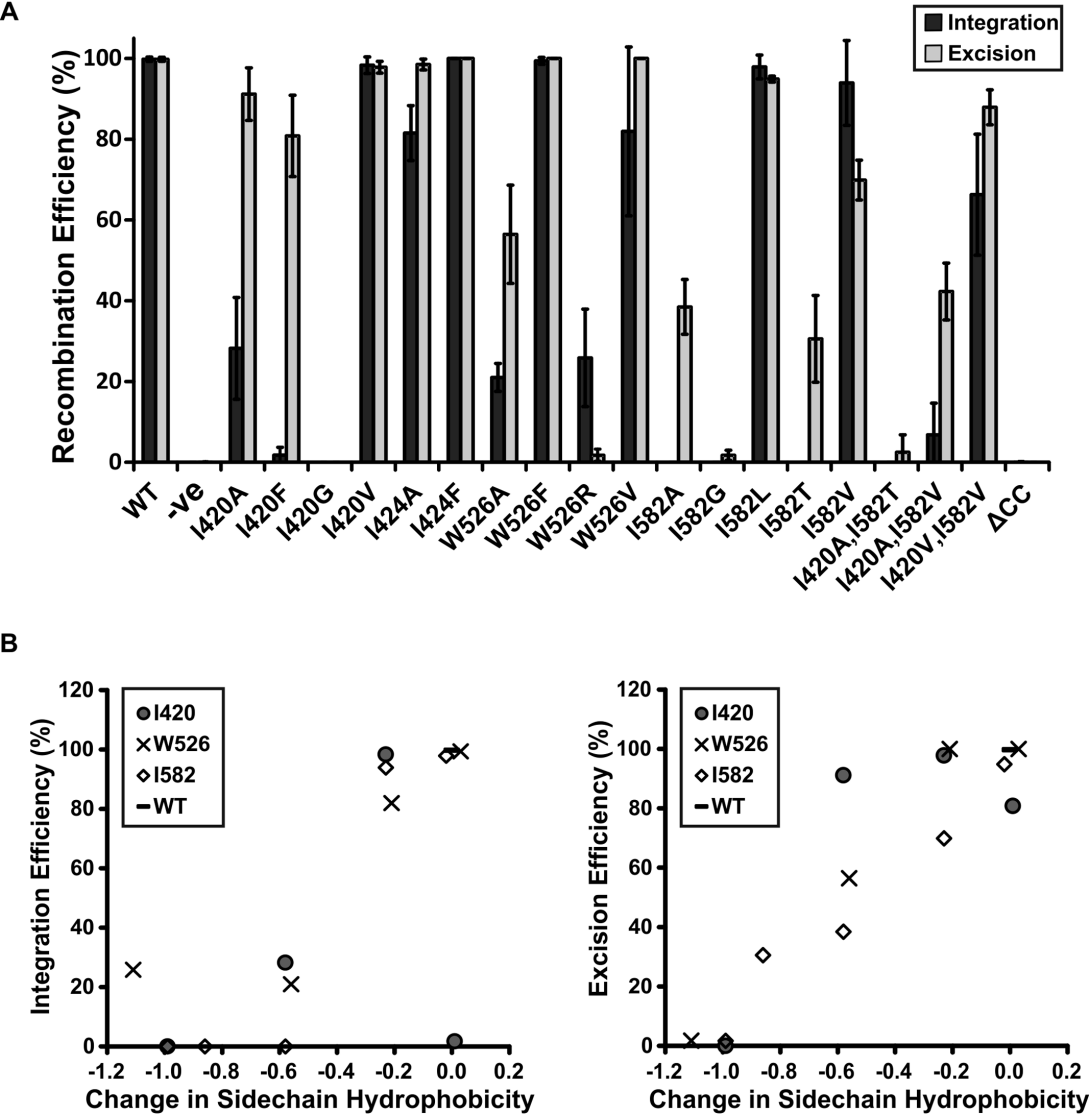
*In vivo* recombination efficiencies of integrase mutants and the effect of amino acid hydrophobicity. (**A**) Histogram of integrase recombination efficiency, defined as the number of white colonies produced as a proportion of the total, after overnight incubation at 37°C. Integration is represented by black bars and excision by gray bars. Mutations tested are labelled on the X-axis and recombination efficiencies on the Y-axis. (**B**) The difference in the normalized hydrophobicity (37) of the wild-type amino acid side-chains compared to those in substitutions is shown on the X-axes against excision or integration efficiency on the Y-axes. White diamonds represent IntI582 mutants; black crosses represent IntW526 mutants; grey circles represent Int I420 mutants and wild-type integrase is the horizontal bar.

Each of the ϕC31 *int* mutations was introduced into pEY4 by site-directed mutagenesis (Supplementary Tables S1 and S2), and these plasmids were then used for *in vivo* intramolecular recombination assays. pEY4 or a derivative encoding a mutant integrase was introduced into *E. coli* containing a reporter plasmid containing *attB-lacZα-attP* (pLT27) to assay integration and *attL-lacZα-attR* (pPAR1000) to assay excision. Excision assays also required a plasmid expressing gp3 (pEY120) (Figure 5). Substitution of any of the amino acids identified above impacted on both the integration and excision activity of ϕC31 integrase; indeed, integration was frequently more severely inhibited (Figure 5A and Supplementary Table S3). This was surprising given that the integrase mutants were identified purely on the basis of their ability to interact with gp3. The conservative substitutions in IntI420V, IntI582L, IntI582V and IntW526F had minimal effect on recombination while in each case a substitution to alanine was significantly more detrimental to integration than excision (Figure 5A and Supplementary Table S3). The only mutants that were more severely reduced in excision over integration were IntW526R and IntI582V. An integrase mutant lacking the coiled-coil motif, IntΔ457–523, was inactive in the *in vivo* recombination assays, which contrasts with the ability of an LI integrase lacking the CC motif to mediate intramolecular *attP* ×*attB* and *attL* ×*attR* recombination (24).

The integration and excision efficiencies for each of the single mutants were plotted against the change in side chain hydrophobicity between the wild-type and mutated residues (Figure 5B and Supplementary Table S4) (36,37). For integration, data points cluster at around 100%, 20% and 0% activity, indicating that the mutated residues have an essentially binary effect on activity (i.e. either the protein is unaffected or it is severely affected). For excision, the plot produces a distinct correlation between recombination efficiency and greater hydrophobicity (Pearson coefficient = 0.91 (37) or 0.85 (36), depending on which hydrophobicity scale is used), which suggests that the RDF is able to mitigate the influence of the mutants to a certain degree. Despite this, the overall picture is that hydrophobic residues located at the base of the coiled-coil motif have an important role in both binding the RDF and in integrative and excisive recombination.

### High concentrations of gp3 can compensate for defective interactions between gp3 and Integrase

**Figure 6.**
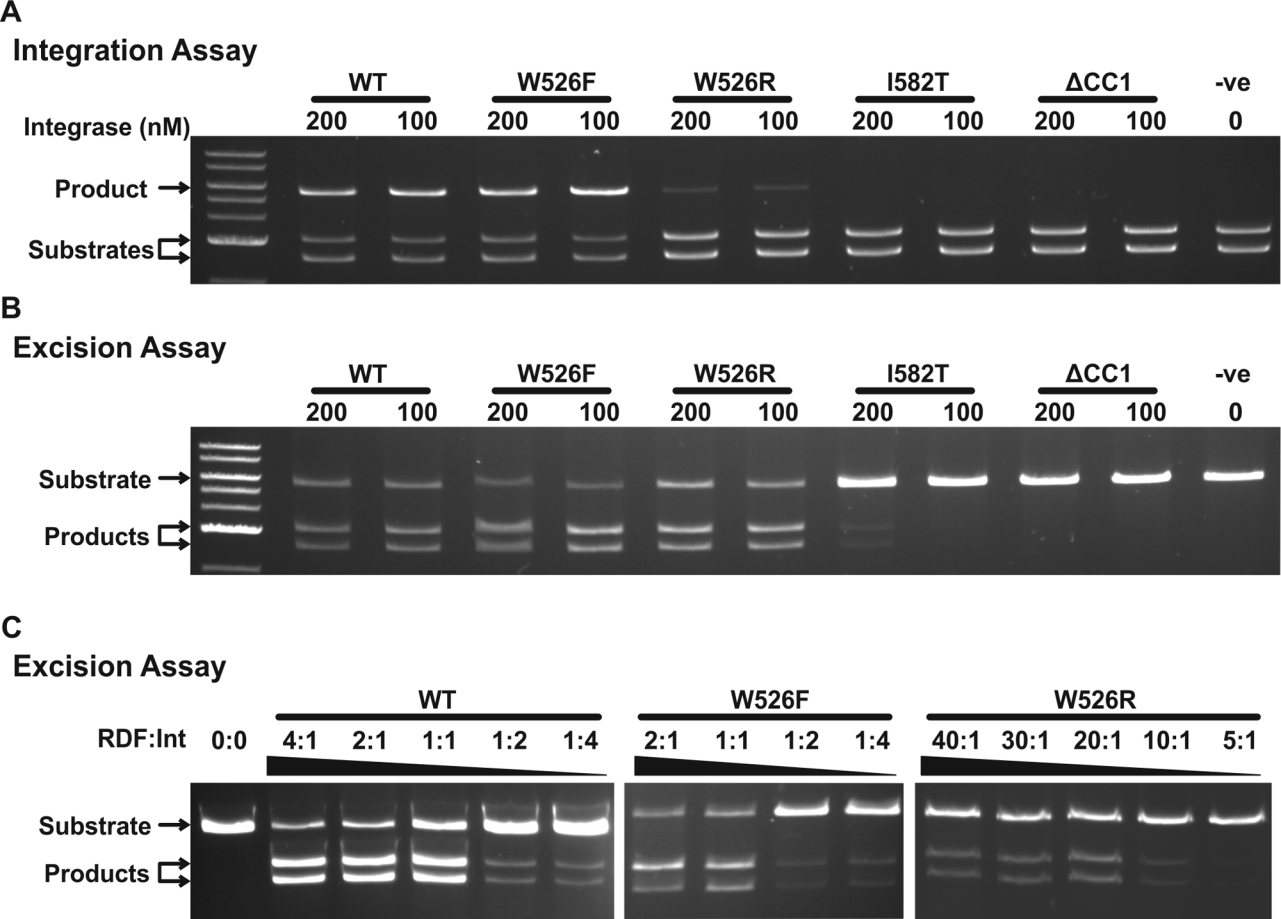
*In vitro* recombination assays of wild-type integrase and mutants. Representative images of *in vitro* integration (**A**) and excision (**B**) assays using wild-type integrase, IntW526F, IntW526R, IntI582T and the coiled-coil deletion (IntΔ457–523) at 100 and 200 nM final concentrations. (**C**) Representative images of *in vitro* excision assays with wild-type RDF titrated against wild-type integrase and two mutant integrases, IntW526R and IntW526F (all integrases at 100 nM).

We sought to validate some of the observed phenotypes from the *in vivo* assays by using purified mutant integrases and gp3s for *in vitro* recombination assays. Several of the ϕC31 integrase mutants (IntW526F, IntW526R, IntI582T and the coiled-coil deletion IntΔ457–523) were overexpressed and purified. For integration, reactions contained the ϕC31 *attB* and *attP* sites located on separate supercoiled plasmids (intermolecular), and integrase (100 or 200 nM). For excision, the reactions contained ϕC31 *attL* and *attR* located head-to-tail on a single supercoiled plasmid (intramolecular), integrase (100 or 200 nM) and an excess of gp3 protein (4500 nM). The coiled-coil deletion mutant IntΔ457–523 was inactive for both integration and excision (Figure 6A and B). The IntI582T mutant was inactive for integration but retained low-level excision activity (Figure 6A and B). The IntW526F mutant was marginally more active than wild-type integrase in both integration and excision reactions (Figure 6A and B). The effect on integration results in an average of 7% extra product (paired-*t P* = 0.0038, *n* = 8) and in excision an average of 15% extra product (paired-*t P* = 0.0004, *n* = 6). Titration of gp3 against IntW526F gives comparable results to experiments with wild-type integrase, i.e. effective excision at equimolar gp3:Int ratio or above (Figure 6C). The *in vitro* recombination activities from these three mutant integrases were, therefore, entirely consistent with the observations from the *in vivo* assays.

IntW526R was only slightly active in the integration reaction, but excision efficiency was approximately equal to that observed with the wild-type integrase (Figure 6A and B). Because the *in vitro* activity for IntW526R contradicted the *in vivo* recombination data, we hypothesized that the large excess of gp3 (22.5-fold) was mitigating the decreased RDF binding affinity of the mutant in this assay, leading to high excision activity. To test this, gp3 was titrated against a constant concentration of integrase (200 nM). Wild-type integrase effectively catalysed excision reactions when gp3 was present at an equimolar ratio or greater, whereas IntW526R was inactive at a 1:1 ratio and only became active when gp3 was in 10- to 20-fold excess (Figure 6C). The ability to compensate for the amino acid substitutions in IntW526R with higher concentrations of gp3 suggests that this substitution leads to a loss of affinity for gp3 rather than a gross change in protein conformation. This is supported by the expression of soluble protein and detectable integration activity. Data consistent with these observations were described above, in which high concentrations of two of the mutant gp3 proteins (gp3M105I and gp3G27E) were able to compensate for their poor activity in excision assays (Figure 1).

## DISCUSSION

**Figure 7.**
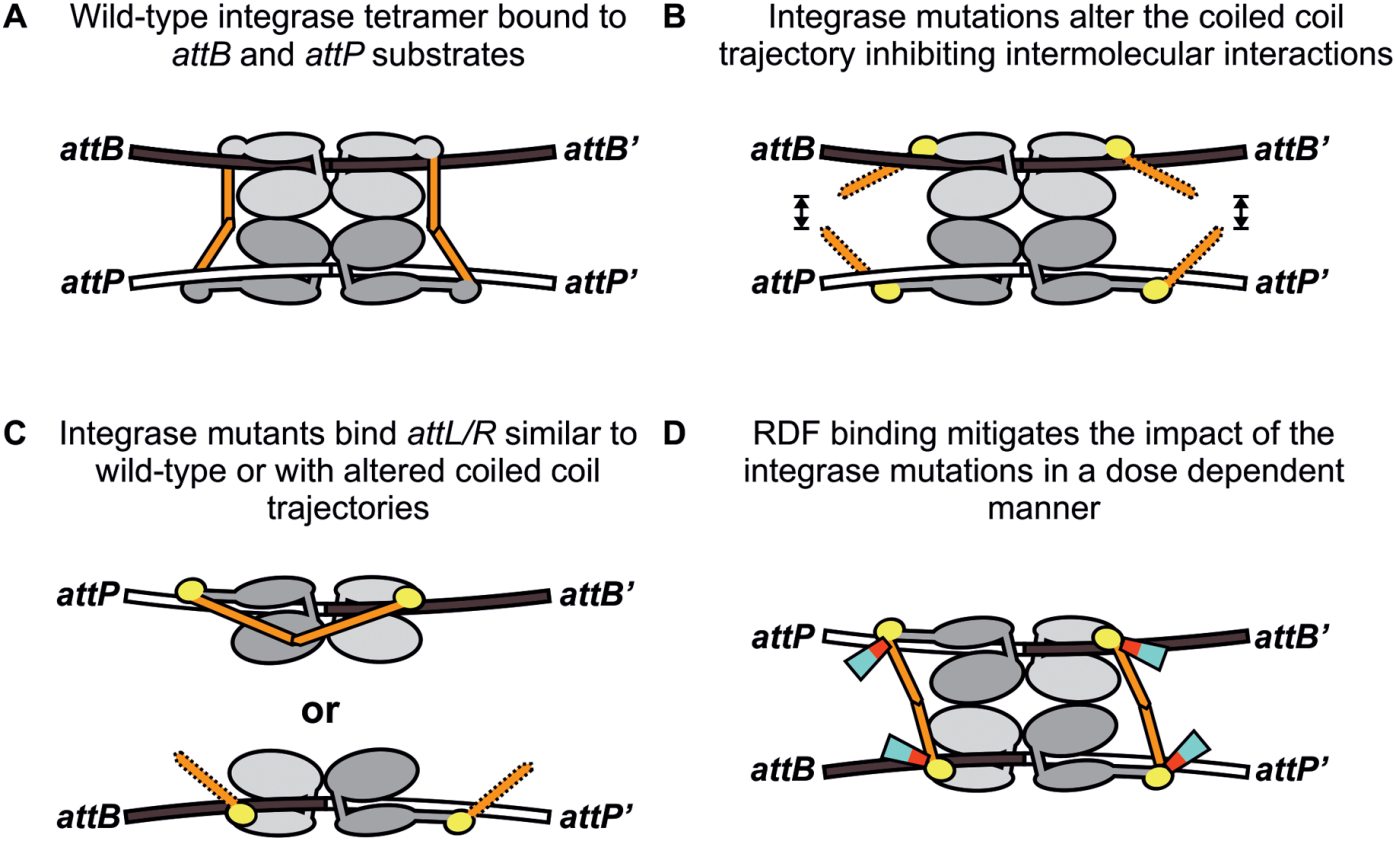
Model of recombination by ϕC31 Int and the effects of coiled-coil mutations. (**A**) Wild-type integrase binds to its *attB* and *attP* sites as two co-operative dimers. The protruding coiled-coil domains interact to stabilize the synaptic complex and to allow recombination to occur. The integrase domain structure depicted is based on recent structures of LI integrase (24,27). The coiled-coil domains are highlighted in orange. (**B**) Single amino acid substitutions were introduced in a putative hinge region (highlighted in yellow) at the base of the coiled-coil motif. The mutations are predicted to alter the trajectory of the coiled-coil, preventing or reducing inter-dimer interactions necessary for efficient recombination. (**C**) Similar to wild-type integrase, the mutated integrases are unable to recombine *attL* and *attR* i.e. no detectable hyperactivity (data not shown). This could be due to WT-like intra-dimer interactions that inhibit recombination, or the coiled-coil motifs may simply be trapped in a non-productive trajectory, similar to the scenario depicted in panel B. (**D**) Binding of the RDF at the base of the coiled-coil motif can reposition it in a way that allows recombination to proceed, and the efficiency with which the RDF can do this is dependent upon binding affinity. We identified seven amino acid substitutions in the RDF that reduce its ability to bind the integrase protein and activate excision. All of the mutated sites are well conserved and six are clustered in a postulated 7 kDa central binding interface, shown in red with the remaining sequence in cyan.

The mechanism by which serine integrases exert directionality was poorly understood until the first structural model was revealed in 2013 (24). The model describes the large C-terminal domain of the *Listeria innocua* (LI) prophage serine integrase (almost identical to phage A118 integrase) bound to its *attP* half-site, and the insights generated from this structure are arguably generally applicable to all serine integrases (25). Central to directional control is the presence of a coiled-coil motif within the zinc domain (ZD) of the protein that has been shown to mediate integrase subunit-subunit interactions (26,27). A general feature of serine integrases is that, in the absence of the RDF, only integrase dimers bound to *attP* and *attB* sites are brought together in the tetrameric synapse. It is widely thought that interactions between the integrase coiled-coils, projecting away from the bound dimers, mediate tetramer formation (24,25,27) (Figure 7A). When integrase dimers are bound to other pairs of attachment sites, the coiled-coil motifs are unable to form inter-dimer interactions, explaining the uniqueness of the *attP* and *attB* sites and their activity in integration. While the coiled-coil motifs in integrase dimers bound to *attL* and *attR* sites should be geometrically able (according to the structure-based model) to interact to generate tetramers, there is an intra-dimer subunit-subunit interaction by the coiled-coil motifs that prevents them from forming tetramers (27,29) (Figure 7C). The crystal structures solved by Rutherford *et al.* show that the coiled-coil motif has a variety of trajectories and this flexibility permits both the proposed inter-dimer interactions (that form tetramers) and the intra-dimer interactions (27). The RDF activates *attL* and *attR* interactions by enabling the formation of the tetrameric synapse for excision (8,19,35,38). The RDF also inhibits *attP* ×*attB* recombination. Data presented here indicate that the ϕC31 RDF, gp3, binds to the ϕC31 integrase ZD at the base of the coiled-coil motif, suggesting that it can direct the trajectory of this interaction motif and therefore influence synapsis of *attP/attB* or *attL/attR* sites. This is consistent with the ideas presented by Rutherford *et al.* on the mechanism of RDFs in activating excision (28) (Figure 7D).

We have shown here that amino acid substitutions in gp3 that lead to defective excision are also defective in binding to integrase, confirming that binding is necessary for activation of excision. Using fragments of integrase in the B2H assay we showed that only the C-terminal 404–605 amino acids of integrase binds to gp3 (Figure 2B). This fragment of integrase contains the whole coiled-coil motif but not the complete ZD domain (Figure 2A). Within this region three residues were identified by mutagenesis (I420, W526 and I582) to be important in gp3 binding. Although these residues are not close together in the primary protein sequence, they are likely to be close in the folded protein (according to a predicted 3D structure and by analogy to the structure of LI integrase), and this region could be the binding site for gp3 (Figure 3). We showed that IntW526R, which has reduced gp3 binding in the B2H assay, has a very low activity in excision assays, but this activity could be compensated by increasing the amount of gp3 added. This suggests that this mutation is likely to reduce the affinity for gp3 rather than affect folding. By investigating the properties conferred by a variety of substitutions at I420, W526 and I582, the overall picture was one where the hydrophobic nature of these residues appeared to be important for gp3 binding and for recombination (Figure 5B, Supplementary Table S4). This rule appeared to extend also to the interaction between ϕBT1 integrase and the ϕC31 RDF, a combination that had been shown to be active for excision despite the poor conservation between ϕBT1 and ϕC31 integrases (Figure 4) (35). This cross-reactivity of ϕC31 RDF with ϕBT1 integrase may be possible because of the rather non-specific nature of hydrophobic interactions.

Of the seven mutations identified in ϕC31 gp3 that affect binding to ϕC31 integrase, six of these lie within 66 amino acids, a length that is reminiscent of the small RDFs from TP901–1, ϕRv1, ϕJoe, SPBc and A118 (17–21). The substitutions occurred in residues that are highly conserved between ϕC31 gp3 and its homologues (Supplementary Figure S1). Possibly the location of the residues and their conservation point to a small structural motif within this family of RDFs that binds to integrase. The predicted secondary structures of the small RDFs listed above are highly variable and comparison with ϕC31 gp3 did not identify any obvious common features.

Integrase mutants with reduced affinity for gp3 in the bacterial-2-hybrid assay were defective in both excision and integration, not just in excision as expected. Indeed integration was often more severely impaired than excision (Figure 5A and Supplementary Table S3). We propose that the recombination deficiencies are because the amino acid changes alter the ability of integrase to adopt different coiled-coil trajectories that affect integrase subunit interactions (Figure 7B). Previous work has shown that the strength of the coiled-coil motif interactions between dimers is quite weak (26,27). For instance, the isolated CTD from ϕC31 integrase can form inter-dimer interactions, probably via the coiled-coil motifs (26), to produce a tetrameric synaptic complex, although the yield of complexes is lower than that obtained with the catalytically inactive, full length integrase, IntS12A. It was proposed that the formation of these inter-dimer CTD interactions might be a prerequisite for the formation of the N-terminal domain (NTD) synaptic interface that activates recombination (26,39). A noticeable difference between the results presented here for ϕC31 integrase and previous work with LI integrase, is that the ϕC31 Int coiled-coil deletion mutant, IntΔCC, is completely inactive under all conditions tested whereas LI IntΔCC is able to catalyse traces of intramolecular recombination *in vitro* and 14% (*attL* ×*attR*) to 100% (*attB* ×*attP*) intramolecular recombination *in vivo* (24,27). Intermolecular recombination was not detected for either integrase. These differences in activity are likely to reflect the energetic balance between the various integrase subunit interactions that ultimately lead to a productive synapse; i.e. the inter- and intra-dimer interactions between the coiled-coil motifs, the NTD synaptic interfaces and any intra-dimer interactions between the coiled-coil and the recombinase domain (see below). The stability of some or all of these interactions is likely to be affected by the status of the putative hinge region that directs the trajectory of the coiled-coil motif; any increase in the energy barrier (resulting from amino acid changes) to inter-dimer interactions mediated by the coiled-coil motifs is likely to be severely inhibitory for integration (Figure 7B). For excision, binding of the RDF could compensate, through release of binding energy or induced fit, for defects in the ability of mutant integrases to adopt appropriate coiled-coil trajectories for excision (Figure 7D). This hypothesis explains how integration can be affected to a greater degree than excision and can be applied to most of the recombination activities of the mutant integrases described here. The notable exceptions are those integrases with no activity *in vivo* (IntI420G and IntI582G) which could be defective in folding, and IntW526R discussed above.

RDFs activate *attL* ×*attR* recombination and generally also inhibit *attP* ×*attB* recombination. If the RDFs control the trajectories of the coiled-coil motifs, then it is entirely possible that they could both activate excision and inhibit integration through this mechanism. Integrase mutants IntI420A and IntI420F are both severely inhibited for integration (even in the absence of the RDF) whilst promoting wild-type levels of excision. Further studies of these mutants might provide some insight into the mechanism of how RDFs inhibit integration.

The likely region of integrase where gp3 binds is close to where amino acid substitutions were isolated that cause so-called hyperactivity; that is, RDF-independent *attL* ×*attR* recombination activity (29). IntE449K, IntE452K, IntE456K and IntE463K are active in an intramolecular *attL* ×*attR* recombination assay whilst remaining highly active in *attP* × *attB* recombination. It has recently been suggested that these mutant integrases might be disrupted in a putative intra-dimer interaction, specifically between the coiled-coil motif and the recombinase domain, when integrase is bound to *attL* or *attR* (27). IntE449K, IntE452K, IntE456K and IntE463K may not therefore be affected in the putative hinge region and this could explain why they are still able to mediate wild-type levels of integration.

Serine integrase RDFs are diverse in size, primary sequence and predicted structure (7,8,17–21). It is unclear at present how RDFs that share little homology with each other are able to perform the same biological role, and no structural information is currently available to make meaningful predictions. Despite this, the available data suggest that all RDFs are likely to act in a similar manner, i.e. by direct interaction with the integrase ZD to promote excision and inhibit integration (19). However, the physical nature of the RDF-integrase interactions is likely to vary substantially among integrase systems.

In summary, mutational analysis of the ϕC31 RDF gp3 indicates that it promotes excision by direct interaction with the integrase. Analysis of the ϕC31 integrase CTD revealed that the putative binding site for gp3 lies at the base of the integrase coiled-coil motif. This region contains a putative hinge that affects the trajectory of the coiled-coil and hence its ability to mediate subunit-subunit interactions to form the synaptic tetramers necessary for excisive recombination to occur. The amino acids involved in the gp3 interaction all suggest that hydrophobicity is important. The work presented here advances our understanding of how serine integrases and their RDFs interact, and this may allow specific engineering of these proteins for different applications and increase the likelihood of identifying novel RDFs.

## Supplementary Material

Supplementary Data
