# Supplementary material for: Recombination directionality factor gp3 binds ϕC31 integrase via the zinc domain, potentially affecting the trajectory of the coiled-coil motif
Source: Nucleic Acids Res. 2017 Dec 8;46(3):1308–20. doi: 10.1093/nar/gkx1233 (PMC5814800; doi:10.1093/nar/gkx1233)
Supplement: Supplementary Data [file gkx1233_supp.docx]

**Supplementary Figures**


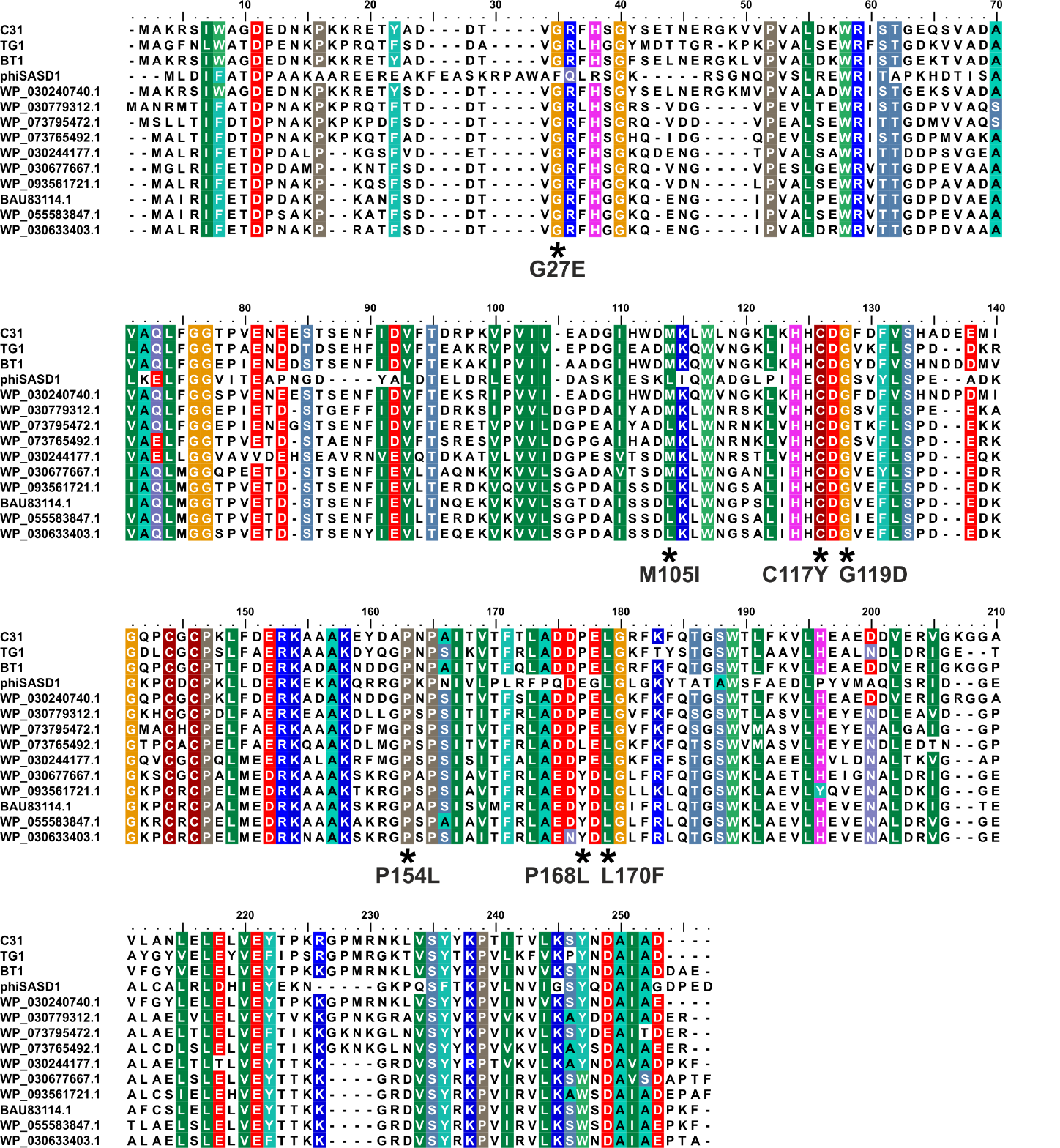


**Figure S1. An extended ClustalΩ alignment of ϕC31 gp3 and related sequences.** The RDF sequences chosen are from all three ϕC31 gp3 homologues that are encoded by verified phage present in GenBank (ϕBT1 gp3, TG1 gp25 and ϕSASD1 gp6) plus the 10 closest Blastp hits to prophage sequences (protein sequence ID is provided as the row titles). All prophage sequences are contained within *Streptomyces* genomes. Shading is based on BLOSUM62 similarity with a 90% threshold. ϕC31 gp3 mutants that affect binding to integrase and excision activity are labelled beneath the alignment.

**
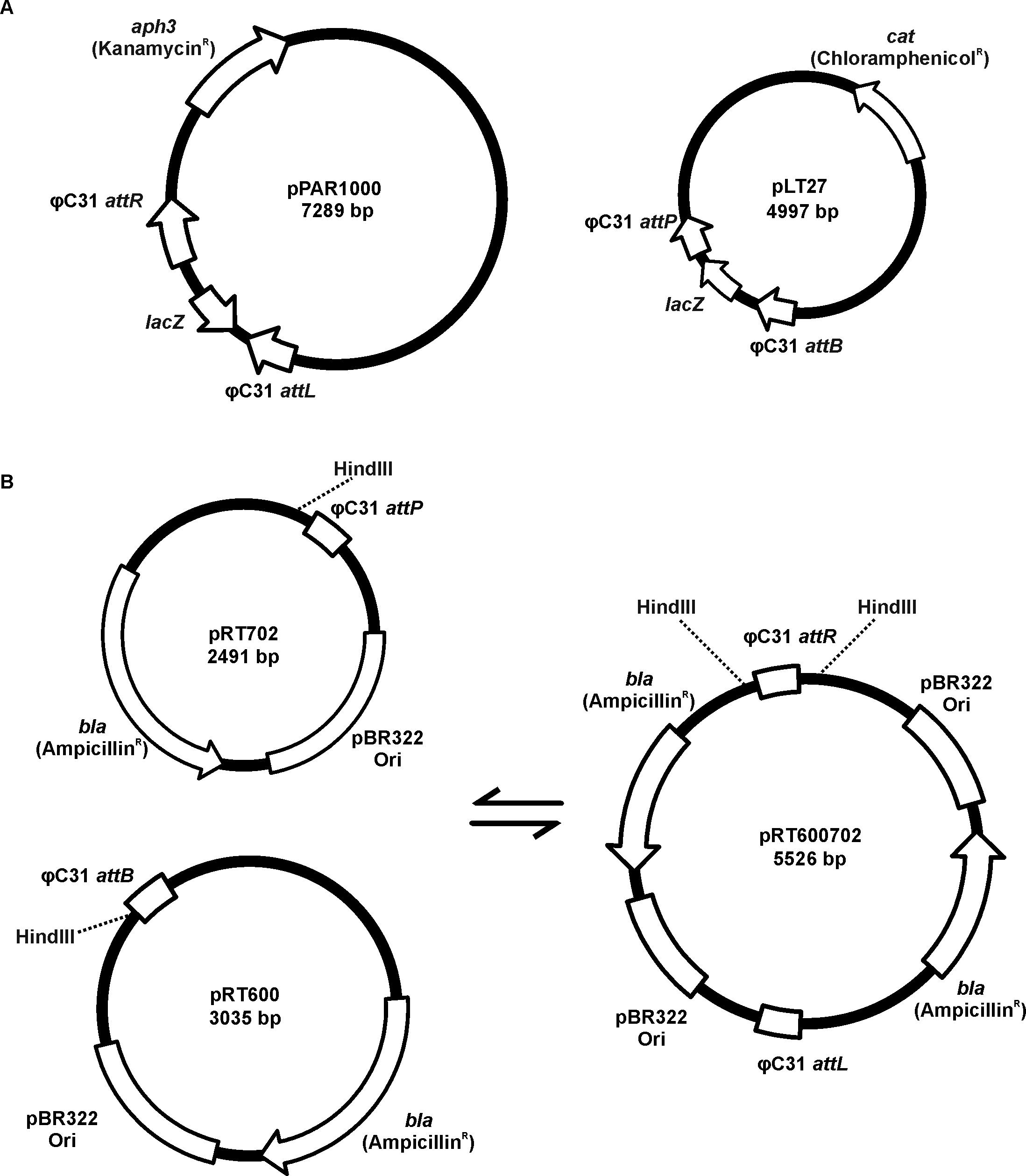
**

**Figure S2. Maps of the plasmids used in recombination assays.** **A.** The *lacZ* reporter plasmids used to assess *in vivo* recombination activities are shown. pPAR1000 contains *attL* and *attR* sites for intramolecular excision assays and pLT27 contains *attB* and *attP* sites for intramolecular integration assays. **B.** *In vitro* integration reactions used two purified plasmids, pRT702 containing ϕC31 *attP* and pRT600 containing ϕC31 *attB*, to assess intermolecular recombination by ϕC31 integrase. The product of the integration reaction, pRT600702 containing ϕC31 *attL* and *attR* sites, was used as a substrate for the intramolecular excision assay. Successful excision by ϕC31 integrase plus gp3 reconstitutes the original *attB* and *attP* plasmids. HindIII sites used to linearize the plasmids are annotated.

**Supplementary Tables**

**Table S1. Full List of Plasmids Used in this Study**

| **Plasmid** | **Description** | **Phage** | **Reference** |
| --- | --- | --- | --- |
| ***In vivo* Recombination Assay** | | | |
| pEY4 | ϕC31 Int, tcp830 promoter | ϕC31 | (Khaleel *et al.*, 2011) |
| pCMF43 | pEY4, Integrase W526R |  | This Study |
| pCMF44 | pEY4, Integrase I582T |  | This Study |
| pCMF57 | pEY4, Integrase W526A |  | This Study |
| pCMF58 | pEY4, Integrase W526F |  | This Study |
| pCMF59 | pEY4, Integrase I582A |  | This Study |
| pCMF60 | pEY4, Integrase I582V |  | This Study |
| pCMF61 | pEY4, Integrase I420A |  | This Study |
| pCMF62 | pEY4, Integrase I420A,I582T |  | This Study |
| pCMF63 | pEY4, Integrase ΔCC1 |  | This Study |
| pCMF79 | pEY4, Integrase I582G |  | This Study |
| pCMF80 | pEY4, Integrase I582L |  | This Study |
| pCMF81 | pEY4, Integrase I582V, I420A |  | This Study |
| pCMF82 | pEY4, Integrase I582V, I420V |  | This Study |
| pCMF83 | pEY4, Integrase I420V |  | This Study |
| pCMF111 | pEY4, Integrase I420F |  | This Study |
| pCMF112 | pEY4, Integrase I424F |  | This Study |
| pCMF113 | pEY4, Integrase I420G |  | This Study |
| pCMF114 | pEY4, Integrase I424A |  | This Study |
| pCMF115 | pEY4, Integrase W526V |  | This Study |
| pARM152 | pACYC-Duet + ϕC31 gp3 |  | This Study |
| pBF01 | pARM152, gp3 L170F |  | This Study |
| pBF02 | pARM152, gp3 M105I |  | This Study |
| pBF03 | pARM152, gp3 G119D |  | This Study |
| pBF13 | pARM152, gp3 G27E |  | This Study |
| pBF14 | pARM152, gp3 P154L |  | This Study |
| pEY120 | pUT18 + ϕC31 gp3 |  | (Khaleel *et al.*, 2011) |
| pBF27 | pEY120, gp3 C117Y |  | This Study |
| pBF64 | pEY120, gp3 P168L |  | This Study |
| pPAR1000 | ϕC31 attL^360^-lacZ-attR^475^ |  | (Rowley *et al.*, 2008) |
| pLT27 | ϕC31 attB^373^-lacZ-attP^464^ |  | (Xu *et al.*, 2013) |
|  |  |  |  |
| **Bacterial 2 Hybrid Plasmids** | | | |
| pKT25 | B2H Vector | ϕC31 | (Karimova *et al.*, 1998) |
| pCMF18 | B2H ϕC31 T25-g3 fusion |  | This Study |
| pCMF7 | pCMF18, g3 L170F |  | This Study |
| pCMF8 | pCMF18, g3 M105I |  | This Study |
| pCMF9 | pCMF18, g3 G119D |  | This Study |
| pCMF11 | pCMF18, g3 G27E |  | This Study |
| pCMF12 | pCMF18, g3 P154L |  | This Study |
| pCMF19 | pCMF18, g3 C117Y |  | This Study |
| pCMF20 | pCMF18, g3 P168L |  | This Study |
| pUT18C | B2H Vector |  | (Karimova *et al.*, 1998) |
| pEY110 | T18-ϕC31 Int fusion (aa 1-605) |  | This study |
| pEY114 | T18-ϕC31 Int fusion (aa 1-203) |  | This study |
| pEY115 | T18-ϕC31 Int fusion (aa 1-407) |  | This study |
| pEY116 | T18-ϕC31 Int fusion (aa 200-605) |  | This study |
| pEY117 | T18-ϕC31 Int fusion (aa 404-605) |  | This study |
| pEY118 | T18-ϕC31 Int fusion (aa 200-407) |  | This study |
| pUT18 | B2H Vector |  | (Karimova *et al.*, 1998) |
| pEY111 | ϕC31 Int-T18 fusion |  | (Khaleel *et al.*, 2011) |
| pCMF36 | pEY111, Integrase D477V |  | This Study |
| pCMF37 | pEY111, Integrase W526R |  | This Study |
| pCMF38 | pEY111, Integrase T564A |  | This Study |
| pCMF39 | pEY111, Integrase I582T |  | This Study |
| pCMF51 | pEY111, Integrase W526A |  | This Study |
| pCMF52 | pEY111, Integrase W526F |  | This Study |
| pCMF53 | pEY111, Integrase I582A |  | This Study |
| pCMF54 | pEY111, Integrase I582V |  | This Study |
| pCMF55 | pEY111, Integrase I420A |  | This Study |
| pCMF56 | pEY111, Integrase I420A,I582T |  | This Study |
| pCMF73 | pEY111, Integrase ΔCC |  | This Study |
| pCMF74 | pEY111, Integrase I582G |  | This Study |
| pCMF75 | pEY111, Integrase I582L |  | This Study |
| pCMF76 | pEY111, Integrase I582V, I420A |  | This Study |
| pCMF77 | pEY111, Integrase I582V, I420V |  | This Study |
| pCMF78 | pEY111, Integrase I420V |  | This Study |
| pCMF119 | pEY111, Integrase W526R, ΔCC1 |  | This Study |
| pCMF46 | pEY111, Integrase I582T, ΔCC1 |  | This Study |
|  |  |  |  |
| pTK32 | B2H ϕBT1 T18-Int fusion | ϕBT1 | This Study |
| pTK33 | B2H ϕBT1 T25-gp3 fusion |  | This Study |
| pCMF120 | pTK32 Integrase W517R |  | This Study |
| pCMF121 | pTK32 Integrase L571T |  | This Study |
|  |  |  |  |
| pCMF30 | B2H TG1 T25-gp25 fusion | TG1 | This Study |
|  |  |  |  |
| **Protein Purification Plasmids for *in vitro* Recombination Assays** | | | |
| pEHISTEV | Expression vector, His6 |  | (Liu and Naismith, 2009) |
| pARM010 | Expression vector, His6-Integrase | ϕC31 | (McEwan *et al.*, 2011) |
| pCMF67 | pARM010, Integrase W526R |  | This Study |
| pCMF71 | pARM010, Integrase I582T |  | This Study |
| pCMF65 | pARM010, Integrase W526F |  | This Study |
| pCMF68 | pARM010, Integrase ΔCC |  | This Study |
| pEY301 | Expression vector, His6-gp3 |  | (Khaleel *et al.*, 2011) |
| pCMF1 | pEHISTEV, gp3 L170F |  | This Study |
| pCMF2 | pEHISTEV, gp3 M105I |  | This Study |
| pCMF3 | pEHISTEV, gp3 G119D |  | This Study |
| pCMF5 | pEHISTEV, gp3 G27E |  | This Study |
| pCMF6 | pEHISTEV, gp3 P154L |  | This Study |
| pRT600 | Recombination substrate, *attB* |  | (Smith *et al.*, 2004) |
| pRT702 | Recombination substrate, *attP* |  | (Smith *et al.*, 2004) |
| pRT600702 | Recombination substrate, *attL & attR* |  | (Smith *et al.*, 2004) |

**Table S2. Oligonucleotides Used in this Study**

| Name | Sequence (5’-3’) | Experiment |
| --- | --- | --- |
| C31 CTD F | ATTCTGTCCGCCATGGACAAGCTGTACTG | Error prone PCR |
| C31 CTD R | CCGGGGATCCTCTAGAGTCGCCGCTACG | Error prone PCR |
| C31 Int I420A 1 | TGCGGAACGCGCCTTCAACAAGATCAGGC | SDM IntI420A |
| C31 Int I420A 2 | CTTGTTGAAGGCGCGTTCCGCAACGAACT | SDM IntI420A |
| C31 Int I420F 1 | TGCGGAACGCTTCTTCAACAAGATCAGGC | SDM IntI420F |
| C31 Int I420F 2 | CTTGTTGAAGAAGCGTTCCGCAACGAACT | SDM IntI420F |
| C31 Int I420G 1 | TGCGGAACGCGGCTTCAACAAGATCAGGC | SDM IntI420G |
| C31 Int I420G 2 | CTTGTTGAAGCCGCGTTCCGCAACGAACT | SDM IntI420G |
| C31 Int I420T 1 | TGCGGAACGCACCTTCAACAAGATCAGGC | SDM IntI420T |
| C31 Int I420T 2 | CTTGTTGAAGGTGCGTTCCGCAACGAACT | SDM IntI420T |
| C31 Int I420V 1 | TGCGGAACGCGTCTTCAACAAGATCAGGC | SDM IntI420V |
| C31 Int I420V 2 | CTTGTTGAAGACGCGTTCCGCAACGAACT | SDM IntI420V |
| C31 Int I424A 1 | CTTCAACAAGGCCAGGCACGCCGAAGGC | SDM IntI424A |
| C31 Int I424A 2 | GCGTGCCTGGCCTTGTTGAAGATGCGTT | SDM IntI424A |
| C31 Int I424F 1 | CTTCAACAAGTTCAGGCACGCCGAAGGC | SDM IntI424F |
| C31 Int I424F 2 | GCGTGCCTGAACTTGTTGAAGATGCGTT | SDM IntI424F |
| C31 Int D477V 1 | CTGTACGAAGTCCGCGCGGCAGGCGCGTAC | SDM IntD477V |
| C31 Int D477V 2 | TGCCGCGCGGACTTCGTACAGCTCTTCAAG | SDM IntD477V |
| C31 Int W526A 1 | CCTTGACCAAGCGTTCCCCGAAGACGCCG | SDM IntW526A |
| C31 Int W526A 2 | TCGGGGAACGCTTGGTCAAGGGGAAGCTTC | SDM IntW526A |
| C31 Int W526F 1 | CCTTGACCAATTCTTCCCCGAAGACGCCG | SDM IntW526F |
| C31 Int W526F 2 | TCGGGGAAGAATTGGTCAAGGGGAAGCTTC | SDM IntW526F |
| C31 Int W526R 1 | CCTTGACCAACGGTTCCCCGAAGACGCCG | SDM IntW526V |
| C31 Int W526R 2 | TCGGGGAACCGTTGGTCAAGGGGAAGCTTC | SDM IntW526R |
| C31 Int W526V 1 | CCTTGACCAAGTGTTCCCCGAAGACGCCG | SDM IntW526V |
| C31 Int W526V 2 | TCGGGGAACACTTGGTCAAGGGGAAGCTTC | SDM IntW526V |
| C31 Int T564A 1 | GATCGTTGTCGCGAAGTCGACTACGGGCAG | SDM IntT564A |
| C31 Int T564A 2 | GTCGACTTCGCGACAACGATCTTGTCTAC | SDM IntT564A |
| C31 Int I582A 1 | CGCGCTTCGGCCACGTGGGCGAAGCCGCC | SDM IntI582A |
| C31 Int I582A 2 | CGCCCACGTGGCCGAAGCGCGCTTCTCGATG | SDM IntI582A |
| C31 Int I582G 1 | CGCGCTTCGGGCACGTGGGCGAAGCCGCC | SDM IntI582G |
| C31 Int I582G 2 | CGCCCACGTGCCCGAAGCGCGCTTCTCGATG | SDM IntI582G |
| C31 Int I582L 1 | CGCGCTTCGCTCACGTGGGCGAAGCCGCC | SDM IntI582L |
| C31 Int I582L 2 | CGCCCACGTGAGCGAAGCGCGCTTCTCGATG | SDM IntI582L |
| C31 Int I582T 1 | CGCGCTTCGACCACGTGGGCGAAGCCGCC | SDM IntI582T |
| C31 Int I582T 2 | CGCCCACGTGGTCGAAGCGCGCTTCTCGATG | SDM IntI582T |
| C31 Int I582V 1 | CGCGCTTCGGTCACGTGGGCGAAGCCGCC | SDM IntI582V |
| C31 Int I582V 2 | CGCCCACGTGACCGAAGCGCGCTTCTCGATG | SDM IntI582V |
| C31 delta CC 1 | GGGGAACCATTGGTCTTCGCCGCTCTTCTCAGG | SDM CC deletion |
| C31 delta CC 2 | GACCAATGGTTCCCCGAAGACGCCGACGCTGAC | SDM CC deletion |
| C31 H6-gp3 F | CTGACCATGGCGAAGCGTTCGATC | pCMF1 to pCMF6 |
| C31 H6-gp3 R | GCAAGCTTGGTGCTCGAGTC | pCMF1 to pCMF6 |
| C31 T25-gp3 F | CGACTCTAGAGGATCCTATGGCGAAGCGTTCGAT | Plasmids derived from pARM152 & pEY120 templates |
| C31 T25-gp3 R | AGGTACCCGGGGATCCAAGCTTGGTGCTCGAGTC | Plasmids derived from pARM152 templates |
| C31 T25-gp3 R2 | AGGTACCCGGGGATCCCCAGGGATCCGCTAGTC | Plasmids derived from pEY120 templates |
| BT1 Int I571T 1 | CCGTGTGACGACCAAGTGGGCGGAGCTGC | pCMF121 |
| BT1 Int I571T 2 | CGCCCACTTGGTCGTCACACGGTCCTTCAG | pCMF121 |
| BT1 Int W517R 1 | TCCGTCTGAGCGGTTCAGCGGCGAAGACC | SDM BTIntW517R |
| BT1 INT W517R 2 | CCGCTGAACCGCTCAGACGGAACGCGGGTG | BTIntW517R |
| TK89 | GCAGTGGAACGCCACTGCAGGATGTCGCCGTTCATCGCTCCGG | pTK32 |
| TK90 | CGGTACCCGGGGATCCCTACTACAGCGCCGCAAGCTCACG | pTK32 |
| EY44 | TAGTGGATCCGATGGCGAAGCGTTCGATCTG | pTK33 |
| TK65 | TAGTGGATCCGCTCACTCGGCGTCGTCGGCAA | pTK33 |
| TG1 T25-RDF F | CGACTCTAGAGGATCCCATGGCCGGATTCAACC | pCMF30 |
| TG1 T25-RDF R | AGGTACCCGGGGATCCAGCTTCGCCATTAGTCG | pCMF30 |
| OARM110 | AGGAGATATACCATGGCATATGTCGTACTACC | pARM121 |
| OARM111 | ATGCGGCCGCAAGCTTGGTGCTCGAGTCAGTC | pARM121 |
| EY94 | ATGCCTGCAGGGCATATGGACACGTACGCGGGTG | pEY110, pEY114. pEY115, |
| EYB2H2v2 | GATCCTCTAGAGTCGCCGCTACGTCTTCCGTGC | pEY110, pEY116, pEY117 |
| Rev4 | ATCCTCTAGAGTAGTGGTCGAGTGCGC | pEY114 |
| Rev3 | ATCCTCTAGAGTGACGTTGCACGTGCC | pEY115, pEY118 |
| For1 | TGCCTGCAGGCACTCGACCACTCCC | pEY116, pEY118 |
| For2 | TGCCTGCAGGACGTGCAACGTCAGC | pEY117 |

**Table S3 –Summary of bacterial 2 hybrid results and *in vivo* recombination efficiencies for all integrase mutants tested**

| **Mutation** | **Bacterial 2 Hybrid** | **Integration (%)** | **n** | **Excision (%)** | **n** |
| --- | --- | --- | --- | --- | --- |
| Wild-type | 100% | 99.8 (SD 0.5) | 7 | 99.8 (SD 0.5) | 7 |
| Control | 0% | 0.0 (SD 0.0) | 4 | 0.0 (SD 0.1) | 5 |
| I420A | 13% | 28.2 (SD 12.6) | 4 | 91.2 (SD 6.5) | 6 |
| I420F | Not Tested | 1.8 (SD 1.9) | 4 | 80.8 (SD 10.1) | 3 |
| I420G | Not Tested | 0.0 (SD 0.0) | 2 | 0.0 (SD 0.0) | 4 |
| I420V | 89% | 98.3 (SD 2.0) | 4 | 97.8 (SD 1.5) | 4 |
| I424A | Not Tested | 81.5 (6.8) | 4 | 98.5 (SD 1.3) | 3 |
| I424F | Not Tested | 100.0 (0.0) | 4 | 100.0 (SD 0.0) | 4 |
| S454G | Positive^ | Not Tested | - | Not Tested | - |
| E476G | Positive^ | Not Tested | - | Not Tested | - |
| D477V | Positive^ | Not Tested | - | Not Tested | - |
| W526A | 16% | 21.0 (SD 3.5) | 4 | 56.4 (SD 12.2) | 5 |
| W526F | 149% | 99.4 (SD 0.8) | 3 | 100.0 (SD 0.0) | 3 |
| W526R | 6% | 25.9 (SD 12.1) | 4 | 1.7 (SD 1.5) | 3 |
| W526V | Not Tested | 81.9 (SD 20.9) | 4 | 100.0 (SD 0.0) | 4 |
| T564A | Positive^ | Not Tested | - | Not Tested | - |
| I582A | 19% | 0.0 (SD 0.0) | 3 | 38.5 (SD 6.8) | 4 |
| I582G | 27% | 0.0 (SD 0.0) | 4 | 1.7 (SD 1.2) | 4 |
| I582L | 104% | 97.9 (SD 2.9) | 4 | 94.9 (SD 0.7) | 3 |
| I582T | 47% | 0.0 (SD 0.0) | 3 | 30.6 (SD 10.7) | 4 |
| I582V | 73% | 93.9 (SD 10.5) | 3 | 69.9 (SD 4.9) | 4 |
| I420A, I582T | -6% | 0.0 (SD 0.0) | 3 | 2.5 (SD 4.3) | 3 |
| I420A, I582V | -11% | 6.8 (SD 7.9) | 4 | 42.3 (SD 7.0) | 3 |
| I420V, I582V | 15% | 66.3 (SD 15.0) | 6 | 87.9 (SD 4.3) | 4 |
| ΔCC | Negative^ | 0.1 (SD 0.1) | 5 | 0.0% (SD 0.0%) | 3 |
| W526R, ΔCC | Negative^ | Not Tested | - | Not Tested | - |
| I582T, ΔCC | Negative^ | Not Tested | - | Not Tested | - |

**^ Qualitative result from plate assay**

**Table S4 – Integrase mutants with *in vivo* recombination efficiencies (Exc. & Integ.), amino acid hydrophobicity changes (ΔM/ΔHH) and Pearson correlations.**

|  | Exc. | ΔHH^34^ | ΔM^33^ | Pearson^ | Integ. | ΔHH^34^ | ΔM^33^ | Pearson^ |
| --- | --- | --- | --- | --- | --- | --- | --- | --- |
| Wild-type | 100% | 0.00 | 0.00 | - | 100% | 0.00 | 0.00 | - |
| I420G | 0.0% | -1.34 | -0.99 |  | 0.0% | -1.34 | -0.58 |  |
| I420A | 91.2% | -0.71 | -0.58 |  | 28.2% | -0.71 | +0.01 |  |
| I420V | 97.8% | -0.29 | -0.23 |  | 98.3% | -0.29 | -0.99 |  |
| I420F | 80.8% | -0.28 | 0.01 | 0.78/0.90 | 1.8% | -0.28 | -0.23 | 0.31/0.51 |
| I424A | 98.5% | -0.71 | -0.58 |  | 81.5% | -0.58 | -0.71 |  |
| I424F | 100.0% | -0.28 | 0.01 |  | 100.0% | 0.01 | -0.28 |  |
| W526R | 1.7% | -2.28 | -1.11 |  | 25.9% | -2.28 | -0.56 |  |
| W526A | 56.4% | 0.19 | -0.56 |  | 21.0% | 0.19 | +0.03 |  |
| W526V | 100.0% | 0.61 | -0.21 |  | 81.9% | 0.61 | -1.11 |  |
| W526F | 100.0% | 0.62 | 0.03 | 0.98/0.95 | 99.4% | 0.62 | -0.21 | 0.87/0.64 |
| I582G | 1.7% | -1.34 | -0.99 |  | 0.0% | -1.34 | -0.58 |  |
| I582T | 30.6% | -1.12 | -0.86 |  | 0.0% | -1.12 | -1.09 |  |
| I582A | 38.5% | -0.71 | -0.58 |  | 0.0% | -0.71 | -0.02 |  |
| I582V | 69.9% | -0.29 | -0.23 |  | 93.9% | -0.29 | -0.86 |  |
| I582L | 94.9% | -0.05 | -0.02 | 0.98/0.98 | 97.9% | -0.05 | -0.23 | 0.92/0.90 |
| Pearson |  | 0.85 | 0.91 |  |  | 0.63 | 0.72 |  |

**^** Pearson correlation values provided for each amino acid position. Monera *et al.* (1995)^33^ and Hessa *et al.* (2005)^34^ hydrophobicity scores are presented – ΔM/ΔHH

**References**

Karimova, G., Pidoux, J., Ullmann, A. and Ladant, D. (1998). "A bacterial two-hybrid system based on a reconstituted signal transduction pathway." *Proc Natl Acad Sci U S A* **95**: 5752-5756.

Khaleel, T., Younger, E., McEwan, A. R., Varghese, A. S. and Smith, M. C. (2011). "A phage protein that binds phiC31 integrase to switch its directionality." *Mol Microbiol* **80**: 1450-1463.

Liu, H. and Naismith, J. H. (2009). "A simple and efficient expression and purification system using two newly constructed vectors." *Protein Expr Purif* **63**: 102-111.

McEwan, A. R., Raab, A., Kelly, S. M., Feldmann, J. and Smith, M. C. M. (2011). "Zinc is essential for high-affinity DNA binding and recombinase activity of φC31 integrase " *Nucleic Acids Res* **39**: 6137-6147.

Rowley, P. A., Smith, M. C., Younger, E. and Smith, M. C. (2008). "A motif in the C-terminal domain of phiC31 integrase controls the directionality of recombination." *Nucleic Acids Res* **36**: 3879-3891.

Smith, M. C. A., Till, R. and Smith, M. C. M. (2004). "Switching the polarity of a bacteriophage integration system." *Mol Microbiol* **51**: 1719-1728.

Xu, Z., Thomas, L., Davies, B., Chalmers, R., Smith, M. and Brown, W. (2013). "Accuracy and efficiency define Bxb1 integrase as the best of fifteen candidate serine recombinases for the integration of DNA into the human genome." *BMC Biotechnol* **13**: 87.
